# Development and validation of a prediction model for serious infections in rheumatoid arthritis patients treated with tocilizumab in Japan

**DOI:** 10.1007/s10067-025-07328-9

**Published:** 2025-02-07

**Authors:** Toshihiro Nanki, Tomohiro Yamaguchi, Kosei Umetsu, Ryunosuke Tanabe, Naoki Maeda, Minori Kanazawa, Yuko Furuno, Shinichi Matsuda, Shinya Takemoto, Keiko Asao, Tatsuya Kamiuchi

**Affiliations:** 1 Division of Rheumatology, Department of Internal Medicine, Toho University School of Medicine, Tokyo, Japan; 2 Drug Safety Division, Chugai Pharmaceutical Co. Ltd, Tokyo, Japan; 3 IQVIA Solutions Japan G.K, Tokyo, Japan

**Keywords:** bDMARDs, IL-6 inhibitor, Prediction model, Rheumatoid arthritis, Serious infections, Tocilizumab

## Abstract

**Objectives:**

To develop a prediction model for serious infections (SIs) in rheumatoid arthritis (RA) patients treated with tocilizumab in Japan and to evaluate the model’s performance compared to previously developed models, i.e., ‘DANBIO’ and ‘postmarketing surveillance’ (PMS).

**Method:**

This non-interventional retrospective cohort study utilized the Medical Data Vision database in Japan. The study population was derived from patients ≥ 18 years with RA who initiated tocilizumab between April 2008 and July 2021. SIs were assessed during the 1-year follow-up from tocilizumab initiation. The candidate predictors were identified based on previous studies, known risk factors, potentially relevant factors, and data availability. The prediction model was developed using logistic regression. The model’s performance was compared with previously developed models using cross-entropy and area under the receiver operating characteristic curve (AUC).

**Results:**

Of the 6501 RA patients, 4.57% experienced SIs during the 1-year follow-up. The model included 17 predictors for SI (e.g., age (odds ratio 1.013 (95% confidence interval 1.002–1.024)), history of SIs (2.569 (1.636–3.745)), diverticulitis (2.183 (1.000–3.989))). The model showed a lower cross-entropy and a higher AUC (0.1488; 0.712) compared to DANBIO (0.1932; 0.591) and PMS (0.1561; 0.565) models, and the sensitivity, specificity, positive predictive value, and negative predictive value using 5% threshold were 72%, 64%, 7%, and 98%, respectively.

**Conclusions:**

The model developed in this study seems to have the potential to inform the risk of SIs in RA patients treated with tocilizumab and may help the early identification of patients at risk of SIs to reduce morbidity and mortality.

**Supplementary Information:**

The online version contains supplementary material available at 10.1007/s10067-025-07328-9.

**Table:** 

**Key Points** •*The developed model in this study may inform physicians on the SIs risk in RA patients treated with tocilizumab.* •*This model can contribute to personalized decision-making, early identification of patients with high-risk profiles to SIs, and reduction in associated morbidity, mortality, and costs.*	

## Introduction

Rheumatoid arthritis (RA) is an autoimmune disease characterized by synovial inflammation and hyperplasia, and progressive destruction of bones and cartilages in multiple joints [1, 2]. RA is associated with increased morbidity, mortality, and socioeconomic burden [2, 3]. The increased morbidity and mortality in patients with RA is in part due to an increased risk of infections [4, 5], which are an important comorbidity in this population [6]. Well-established risk factors for infection in RA patients are disease severity, older age, previous infections, comorbidities, and current immunosuppressive therapy [7–9].

Tocilizumab, an interleukin-6 (IL-6) receptor inhibitor, is a biologic disease-modifying antirheumatic drug (DMARD) for adult patients with moderate to severe, active RA who have had an inadequate response to one or more DMARDs [10–15]. The use of biologic DMARDs (bDMARDs) for the treatment of RA has been associated with a risk of serious infections (SIs) [9]; however, this risk seems to vary across bDMARDs [16, 17]. The evidence regarding the risk of SI in patients treated with tocilizumab is limited. The available evidence suggests a similar rate of SI in tocilizumab-treated RA patients compared with other bDMARDs [17–20].

A few models have been developed to predict infections in patients with RA, including a scoring system by Crowson, et al. [21], the Rheumatoid Arthritis Observation of Biologic Therapy (RABBIT) risk score [22], the nationwide rheumatology registry (DANBIO) infection risk score model [23], a scoring system by Yang, et al. [24], and a model using the postmarketing surveillance (PMS) data of tocilizumab users in Japan (‘PMS model’) [25]. However, the number of patients treated with tocilizumab in those studies was limited. Of these models, Crowson’s scoring system did not specify treatment received by the patients [21], the RABBIT risk score included patients treated with DMARDs and tumor necrosis factor (TNF) inhibitors [22], and Yang’s scoring system included patients treated with first-line methotrexate (MTX) monotherapy [24]. Although the DANBIO and PMS models were developed in RA patients treated with bDMARDs, including tocilizumab [23, 25], the predictive performance of such models in the Japanese population is yet still needed to be validated. The development of a risk prediction model, along with the identification of the main risk factors for SIs in RA patients treated with tocilizumab in the Japanese population, could contribute to personalized decision-making on treatment choice, early identification of patients at high risk of SI, and reduction in associated morbidity, mortality, and subsequently healthcare costs in Japan.

This study aimed to develop a risk prediction model for SIs and to evaluate the performance of the developed prediction model compared to the existing DANBIO and PMS models [23, 25] in RA patients treated with tocilizumab using an administrative healthcare database in Japan.

## Materials and methods

### Study design

A non-interventional retrospective cohort study was conducted using the Medical Data Vision (MDV) database (Medical Data Vision, Co., Ltd. Tokyo, Japan) [26], which is an anonymized database covering approximately 9.7% of the Japanese population and 27.5% of the acute hospitals [27]. Although it does not include some clinical information specific to RA (e.g., findings of tender or swollen joints or laboratory test results), the data has information on demographics, diagnoses, medications, procedures, and hospitalisation from 2008.

The study was conducted in accordance with the Declaration of Helsinki [28] and with the Japanese Ethical Guidelines for Medical and Biological Research Involving Human Subjects. Ethical approval was obtained from the Non-Profit Organisation MINS Research Ethics Committee on 11th January 2023 (MINS-REC-230201). The MDV database was anonymized; thus, informed consent was not obtained from the study participants. The transparent reporting of a multivariable prediction model for individual prognosis or diagnosis (TRIPOD) guidelines was followed [29].

### Patient population

Patients who met the following criteria between 1st April 2008 and 31st July 2021 were included: prescribed the first prescription of tocilizumab (the index date); aged ≥ 18 years at the tocilizumab initiation; having a definitive diagnosis of RA (defined as the International Classification of Diseases, 10th revision (ICD-10) codes M05 and M06) during the 6-month (180-day) baseline period preceding tocilizumab initiation; and a history of at least two antirheumatic drug prescriptions during the baseline period. Patients were excluded if they had no records of tocilizumab prescription after the index date, a history of malignancy treatment during the baseline period or at the index date, no at least 4-month (120 days) lookback period before the index date, and a prescription of tocilizumab for a disease other than RA during the baseline period or at the index date (except suspected disease, i.e., without a definitive diagnosis). For further details, please refer to Supplementary Table S1. The number of patients receiving bDMARDs in the MDV database was assessed before the study; however, no formal sample size calculations were done.

### Outcome and follow-up period

The outcome, i.e., SIs, was defined as a record of inpatient hospitalisation with the diagnosis of infection in the presence of immunological infection tests and other related tests (e.g., antibody tests and antigen tests) within 1 month (30 days) before the date of hospitalization or during the hospitalization (for further details, see Supplementary Table S2) [30]. The definition of SIs was validated to identify SIs in MDV and yielded a positive predictive value of 85.6% [30].

SIs were assessed during the 1-year (365 days) follow-up period from the index to the earliest date of the following censoring criteria: last date of recorded data, last day of the study period (31st July 2022), death, 1 year after the index date, and date of tocilizumab discontinuation (i.e., no records of subsequent tocilizumab prescription for more than 3 months (90 days) after the most recent tocilizumab prescription).

### Baseline characteristics

The baseline characteristics included patient’s demographics (sex and age), RA disease characteristics (duration of RA), history of infections, concomitant disease (constipation), comorbidities (e.g., liver diseases, renal diseases, respiratory diseases, heart diseases, vascular diseases, gastrointestinal diseases, knee or hip prosthesis, diabetes, and malignancies), laboratory data (C-reactive protein [CRP]), co-treatment (DMARDs, MTX, TNF inhibitors, and steroids), and histories of RA treatment (conventional synthetic DMARDs (csDMARDs) and bDMARDs). Information on these characteristics in each patient was collected before or at the index date with the varying time of assessment (e.g., age at the index date, duration of RA during the entire data period until the index date, DMARDs use during 1 week period before the index date). For more information, refer to Supplementary Table S3.

### Predictors of SI

The candidate predictors of SI included variables used in previous studies [22, 23], risk factors reported in the past study [25], and factors considered potentially relevant from clinical perspectives. Some candidates were removed or modified based on the availability of the data in the MDV database. They included baseline variables with varying assessment periods relative to the index date and varying categorizations. For further details, refer to Supplementary Table S3. Predictors of SI in each model were selected from candidate predictors using statistical analysis.

### Statistical analysis

For descriptive analysis, mean (standard deviation [SD]), median (first and third quartiles (Q1, Q3)), and minimum and maximum values were reported for continuous variables, while counts and percentages were reported for categorical variables. Missing values, if any, were reported as a separate category and descriptive statistics were based on non-missing values only.

The prediction model to estimate the probability of SIs in RA patients was developed using a pre-specified list of candidate predictors (Supplementary Table S3). Some candidate predictors were subject to variation in operational definition, e.g., in the assessment period or categorizations (for continuous predictors). Therefore, multiple sets of candidate predictors were considered. Also, predictors were standardized using *z*-score normalization. No missing data was observed in these predictors.

The overall population was split into training and test sets, where the test set was formed from the most recent 20% of index dates. The training set was used for the development of the prediction model. The test set was used for temporal validation and comparison of the developed model with the DANBIO model [23] and PMS model [25].

Least absolute shrinkage and selection operator (LASSO) based on logistic regression was used as a prediction model with the group LASSO approach to handle the categorical variables [31]. This method tends to shrink the coefficients towards zero, resulting in a subset of variables. For each combination of candidate predictors and LASSO penalty value, *λ*, (from the pre-specified list of 16 candidate *λ* values ranging from 0 to 10,000), an average cross-entropy measure from tenfold cross-validation in the training set was calculated to choose the optimal *λ* for each set of candidate predictors. All combinations were ranked based on this average cross-entropy measure and respective LASSO models were fitted in the training set. Ten models were selected: five highest-ranking models without restrictions and five highest-ranking with no more than ten non-zero coefficients. The model with the lowest average cross-entropy measure was denoted as the developed model. The contribution of each predictor to risk prediction was reported using raw estimated predictor coefficients and exponentiated coefficients, representing odds ratios (ORs) with 95% confidence intervals (CIs) obtained using bootstrap method with 1000 replicates.

The performance of prediction models was assessed in the test set using cross-entropy and area under the receiver operating characteristic (ROC) curve (AUC) measures. Furthermore, overall prediction accuracy, sensitivity, specificity, and positive and negative predictive values at different prediction threshold levels (from 5 to 95%) were calculated. In addition, calibration plots were produced displaying the predicted (sum of predicted probabilities) versus observed (proportion of infection outcomes) risk for each decile of predicted risk. DANBIO and PMS models were applied in the test set to compare their performance with the developed model using the measures described above.

All data processing, computations, and generation of tables and figures were performed using R version 4.2.2 [32].

## Results

### Baseline characteristics

A total of 6501 RA patients were included in the overall population with a mean follow-up of 292.10 (SD 101.65) days. In the overall population (Table 1), the mean (SD) age was 63.70 (SD 13.76) years, most were females (75.65%) and the mean duration of RA was 2.29 (SD 2.09) years. Most patients (87.31%) had a history of infection prior to the index date and 4.81% had a history of SI within 1 year before the index date. The most frequent comorbidities (assessed at any time before the index date) were respiratory disease (66.41%) and vascular disease (43.30%). In total, 23.12% had a history of malignancy within 10 years before the index date. Regarding co-treatment, 65.57% had used any DMARDs and 43.95% MTX within 1 week before the index date. Steroids were used by 53.48% of the patients within 1 week before the index date and by 72.39% of the patients within 1 year before the index date. Further details, including the baseline characteristics of the training and test sets, can be found in Table 1.

**Table 1:** Baseline characteristics of patients with rheumatoid arthritis treated with tocilizumab

Variable	Overall population (*N* = 6501)	Training set (*N* = 5199)	Test set (*N* = 1302)
**Demographics**			
Sex (*n* (%))			
Male	1583 (24.35%)	1248 (24.00%)	335 (25.73%)
Female	4918 (75.65%)	3951 (76.00%)	967 (74.27%)
Age (years)			
Mean (SD)	63.70 (13.76)	63.23 (13.67)	65.61 (13.97)
Median	66.00	65.00	68.00
Q1, Q3	55.00, 74.00	55.00, 73.00	57.00, 76.00
Min, Max	18, 96	18, 96	20, 92
Age (years) (*n* (%))			
< 40	395 (6.08%)	330 (6.35%)	65 (4.99%)
40–49	652 (10.03%)	541 (10.41%)	111 (8.53%)
50–59	1144 (17.60%)	931 (17.91%)	213 (16.36%)
60–69	1814 (27.90%)	1509 (29.02%)	305 (23.43%)
≥ 70	2496 (38.39%)	1888 (36.31%)	608 (46.70%)
**RA disease characteristics**			
Duration of RA disease (years)			
Mean (SD)	2.29 (2.09)	2.19 (1.96)	2.69 (2.51)
Median	1.58	1.54	1.84
Q1, Q3	0.70, 3.28	0.70, 3.13	0.66, 4.09
Min, Max	0.01, 12.87	0.01, 12.05	0.01, 12.87
Duration of RA disease (years) (*n* (%))			
< 10	6471 (99.54%)	5184 (99.71%)	1287 (98.85%)
≥ 10	30 (0.46%)	15 (0.29%)	15 (1.15%)
**History of infections,** *n* (%)			
Infections, ever	5676 (87.31%)	4551 (87.54%)	1125 (86.41%)
Serious infections within 1 year	313 (4.81%)	260 (5.00%)	53 (4.07%)
Serious infections within 5 years	603 (9.28%)	470 (9.04%)	133 (10.22%)
**Concomitant disease,** *n* (%)			
Constipation within the same month as the index date	1290 (19.84%)	1025 (19.72%)	265 (20.35%)
**Comorbidities, ***n* (%)			
Liver diseases, ever	1071 (16.47%)	830 (15.96%)	241 (18.51%)
Renal diseases, ever	928 (14.27%)	731 (14.06%)	197 (15.13%)
Respiratory diseases, ever	4317 (66.41%)	3465 (66.65%)	852 (65.44%)
Heart diseases, ever	1598 (24.58%)	1248 (24.00%)	350 (26.88%)
Vascular diseases, ever	2815 (43.30%)	2228 (42.85%)	587 (45.08%)
Diverticulitis, ever	78 (1.20%)	57 (1.10%)	21 (1.61%)
Amyloidosis, ever	105 (1.62%)	89 (1.71%)	16 (1.23%)
Gastrointestinal ulcers, ever	2219 (34.13%)	1803 (34.68%)	416 (31.95%)
Knee or hip prostheses, ever	271 (4.17%)	216 (4.15%)	55 (4.22%)
Knee or hip prostheses within 5 years	249 (3.83%)	199 (3.83%)	50 (3.84%)
Interstitial lung diseases, ever	1783 (27.43%)	1430 (27.51%)	353 (27.11%)
Interstitial lung diseases within 5 years	1773 (27.27%)	1425 (27.41%)	348 (26.73%)
Diabetes, ever	2347 (36.10%)	1837 (35.33%)	510 (39.17%)
Diabetes within 5 years	2329 (35.83%)	1826 (35.12%)	503 (38.63%)
Myocardial infarction, ever	42 (0.65%)	32 (0.62%)	10 (0.77%)
Myocardial infarction within 5 years	41 (0.63%)	31 (0.60%)	10 (0.77%)
Chronic kidney disease, ever	350 (5.38%)	262 (5.04%)	88 (6.76%)
Chronic kidney diseases within 5 years	350 (5.38%)	262 (5.04%)	88 (6.76%)
IBD, ever	76 (1.17%)	65 (1.25%)	11 (0.84%)
IBD within 5 years	75 (1.15%)	65 (1.25%)	10 (0.77%)
Malignancies^a^, ever	1503 (23.12%)	1130 (21.73%)	373 (28.65%)
Malignancies^a^ within 10 years	1503 (23.12%)	1130 (21.73%)	373 (28.65%)
**Laboratory data, within 2 weeks**			
CRP (mg/dL)			
N	430	345	85
Mean (SD)	3.03 (3.40)	3.06 (3.42)	2.92 (3.38)
Median	1.77	1.90	1.61
Q1, Q3	0.45, 4.62	0.49, 4.62	0.32, 4.19
Min, Max	0.01, 24.51	0.01, 24.51	0.01, 16.32
Missing	6071 (93.39%)	4854 (93.36%)	1217 (93.47%)
**Co-treatment, ***n* (%)			
DMARDs within 1 week	4263 (65.57%)	3389 (65.19%)	874 (67.13%)
MTX within 1 week	2857 (43.95%)	2301 (44.26%)	556 (42.70%)
Salazosulfapyridine within 1 week	1271 (19.55%)	959 (18.45%)	312 (23.96%)
Leflunomide within 1 week	36 (0.55%)	30 (0.58%)	6 (0.46%)
TNF inhibitor/other biologics within 1 week	18 (0.28%)	13 (0.25%)	5 (0.38%)
Oral steroids, within 1 week	3319 (51.05%)	2656 (51.09%)	663 (50.92%)
Injection steroids, within 1 week	352 (5.41%)	280 (5.39%)	72 (5.53%)
Any steroids within 1 week	3477 (53.48%)	2780 (53.47%)	697 (53.53%)
Any steroids within 1 year	4706 (72.39%)	3797 (73.03%)	909 (69.82%)
Steroids (mg/day) within 1 week			
0	3030 (46.61%)	2422 (46.59%)	608 (46.70%)
0–5	1845 (28.38%)	1477 (28.41%)	368 (28.26%)
> 5	1626 (25.01%)	1300 (25.00%)	326 (25.04%)
**History of treatment, ***n* (%)			
Number of previous treatments with DMARDs, ever			
> 5	116 (1.78%)	78 (1.50%)	38 (2.92%)
≤ 5	6385 (98.22%)	5121 (98.50%)	1264 (97.08%)
Number of previous treatments with csDMARDs, ever			
0	562 (8.64%)	451 (8.67%)	111 (8.53%)
1	2739 (42.13%)	2243 (43.14%)	496 (38.10%)
2	1943 (29.89%)	1549 (29.79%)	394 (30.26%)
3	921 (14.17%)	713 (13.71%)	208 (15.98%)
≥ 4	336 (5.17%)	243 (4.67%)	93 (7.14%)

*Abbreviations CRP* C-creative protein, *csDMARD* conventional synthetic disease-modifying antirheumatic drug, *DMARD* disease-modifying antirheumatic drug, *IBD* inflammatory bowel diseases, *Max* maximum, *Min* minimum, *MTX* methotrexate, *Q1* first quartile, *Q3* third quartile, *RA* rheumatoid arthritis, *SD* standard deviation, *TNF* tumor necrosis factor; The variables were assessed before or at the index date for the varying time of assessment. For more information, please refer to Supplementary Table S3. Summary statistics and % were calculated by excluding missing data. Missing values were reported only when observed and applicable. In multiple observations, the value closest to the index date was used (where applicable). The training set is a subset of the full analysis set (80% of the overall population) to train the predictive models. The test set a subset of the full analysis set (the latest 20% of eligible patients of the dataset) to evaluate the predictive performance of the models

^a^Only patients without active malignancy treatment on the index date were considered

### Censored patients

In the overall population, 95.43% (*n* = 6204) of the RA patients were censored at the end of the study, with similar distributions between training (*n* = 4948, 95.17%) and test (*n* = 1256, 96.47%) sets. Reasons for end of follow-up were exit from database (overall population *n* = 354, 5.45%; training set *n* = 178, 3.42%; and test set *n* = 176, 13.52%) and other (i.e., end of 1-year follow-up, death, and tocilizumab discontinuation) (overall population *n* = 5850, 89.99%; training set *n* = 4770, 91.75%; and test set *n* = 1080, 82.95%).

### Occurrence of serious infections

In the overall population, 4.57% (*n* = 297) had the occurrence of SI during follow-up. The most frequent SIs were influenza and pneumonia (*n* = 54, 0.83%), infections of the skin and subcutaneous tissue (*n* = 38, 0.58%), and other bacterial diseases (*n* = 21, 0.32%). More details, including the occurrence of SI events between the training and test sets, can be found in Table 2. The frequency of SIs by ICD-10 codes is presented in Supplementary Table S4.

**Table 2:** The numbers and proportions of patients with rheumatoid arthritis treated with tocilizumab with serious infections

ICD-10	Disease name	Overall population (*N* = 6501)	Training set (*N* = 5199)	Test set (*N* = 1302)
Any serious infections, *n* (%)		297 (4.57%)	251 (4.83%)	46 (3.53%)
Serious infections by category***, ****n* (%)				
A00–A09	Intestinal infectious diseases	15 (0.23%)	13 (0.25%)	2 (0.15%)
A30-–A49	Other bacterial diseases	21 (0.32%)	15 (0.29%)	6 (0.46%)
A50–A64	Infections with a predominantly sexual mode of transmission	1 (0.02%)	1 (0.02%)	0 (0.00%)
B00–B09	Viral infections characterized by skin and mucous membrane lesions	16 (0.25%)	15 (0.29%)	1 (0.08%)
B15–B19	Viral hepatitis	1 (0.02%)	1 (0.02%)	0 (0.00%)
B25–B34	Other viral diseases	1 (0.02%)	1 (0.02%)	0 (0.00%)
B35–B49	Mycoses	4 (0.06%)	4 (0.08%)	0 (0.00%)
G00–G09	Inflammatory diseases of the central nervous system	2 (0.03%)	2 (0.04%)	0 (0.00%)
G50–G59	Nerve, nerve root, and plexus disorders	1 (0.02%)	1 (0.02%)	0 (0.00%)
G60–G64	Polyneuropathies and other disorders of the peripheral nervous system	1 (0.02%)	1 (0.02%)	0 (0.00%)
H15–H22	Disorders of sclera, cornea, iris, and ciliary body	2 (0.03%)	2 (0.04%)	0 (0.00%)
H30–H36	Disorders of choroid and retina	3 (0.05%)	3 (0.06%)	0 (0.00%)
H65–H75	Diseases of middle ear and mastoid	1 (0.02%)	1 (0.02%)	0 (0.00%)
H80–H83	Diseases of inner ear	5 (0.08%)	5 (0.10%)	0 (0.00%)
I30–I52	Other forms of heart disease	4 (0.06%)	3 (0.06%)	1 (0.08%)
I70–I79	Diseases of arteries, arterioles, and capillaries	7 (0.11%)	6 (0.12%)	1 (0.08%)
I80–I89	Diseases of veins, lymphatic vessels, and lymph nodes, not elsewhere classified	3 (0.05%)	1 (0.02%)	2 (0.15%)
J09–J18	Influenza and pneumonia	54 (0.83%)	45 (0.87%)	9 (0.69%)
J20–J22	Other acute lower respiratory infections	2 (0.03%)	2 (0.04%)	0 (0.00%)
J30–J39	Other diseases of upper respiratory tract	3 (0.05%)	2 (0.04%)	1 (0.08%)
J40–J47	Chronic lower respiratory diseases	1 (0.02%)	1 (0.02%)	0 (0.00%)
J85–J86	Suppurative and necrotic conditions of lower respiratory tract	2 (0.03%)	1 (0.02%)	1 (0.08%)
J90–J94	Other diseases of pleura	2 (0.03%)	2 (0.04%)	0 (0.00%)
J95–J99	Other diseases of the respiratory system	1 (0.02%)	1 (0.02%)	0 (0.00%)
K20–K31	Diseases of esophagus, stomach, and duodenum	3 (0.05%)	3 (0.06%)	0 (0.00%)
K35–K38	Diseases of appendix	4 (0.06%)	4 (0.08%)	0 (0.00%)
K50–K52	Noninfective enteritis and colitis	1 (0.02%)	1 (0.02%)	0 (0.00%)
K55–K64	Other diseases of intestines	12 (0.18%)	12 (0.23%)	0 (0.00%)
K65–K67	Diseases of peritoneum	6 (0.09%)	5 (0.10%)	1 (0.08%)
K70–K77	Diseases of liver	1 (0.02%)	1 (0.02%)	0 (0.00%)
K80–K87	Disorders of gallbladder, biliary tract, and pancreas	13 (0.20%)	13 (0.25%)	0 (0.00%)
K90–K93	Other diseases of the digestive system	3 (0.05%)	3 (0.06%)	0 (0.00%)
L00–L08	Infections of the skin and subcutaneous tissue	38 (0.58%)	28 (0.54%)	10 (0.77%)
L40–L45	Papulosquamous disorders	1 (0.02%)	1 (0.02%)	0 (0.00%)
L80–L99	Other disorders of the skin and subcutaneous tissue	3 (0.05%)	3 (0.06%)	0 (0.00%)
M00–M25	Arthropathies	11 (0.17%)	10 (0.19%)	1 (0.08%)
M40–M54	Dorsopathies	14 (0.22%)	10 (0.19%)	4 (0.31%)
M60–M79	Soft tissue disorders	2 (0.03%)	2 (0.04%)	0 (0.00%)
M80–M94	Osteopathies and chondropathies	5 (0.08%)	4 (0.08%)	1 (0.08%)
N10–N16	Renal tubulo-interstitial diseases	14 (0.22%)	12 (0.23%)	2 (0.15%)
N30–N39	Other diseases of urinary system	4 (0.06%)	3 (0.06%)	1 (0.08%)
N70–N77	Inflammatory diseases of female pelvic organs	1 (0.02%)	1 (0.02%)	0 (0.00%)
T80–T88	Complications of surgical and medical care, not elsewhere classified	8 (0.12%)	6 (0.12%)	2 (0.15%)

*Abbreviations* *ICD-10* The World Health Organization International Classification of Diseases 10th Revision; Summary statistics were calculated after excluding missing values. The training set is a subset of the full analysis set (80% of the overall population) to train the predictive models. The test set is a subset of the full analysis set (the latest 20% of eligible patients of the dataset) to evaluate the predictive performance of the models

### Development of prediction model

Ten candidate models with different sets of predictors were developed to predict SI in RA patients treated with tocilizumab using the training set (*N* = 5199) (see supplementary Table S5). All the models performed similarly, with values ranging between 0.1820 and 0.1858 for average cross-validated cross-entropy in the training set, 0.1483 to 0.1507 for cross-entropy in the test set, and 0.686 to 0.716 for AUC. Refer to Supplementary Table S5 for the results of model discrimination at a 5% threshold risk. Other prediction threshold levels were not reported due to low predictive performance.

Of the ten developed candidate models, the model with the lowest average cross-validated cross-entropy (0.1820) was chosen as preferred (referred to as the developed model; Model 1 in Supplementary Table S5). The equation and spread-sheet sample to calculate the individualized probability of SIs is shown in Supplementary Tables S6 and S7, respectively. The developed model included 17 predictors of SIs, including age (OR 1.013 (95% CI 1.002–1.024)), history of previous SIs within 1 year (OR 2.569 (1.636–3.745)), diverticulitis and related disorders (OR 2.183 (1.000–3.989)), heart disease (OR 1.391 (1.006–1.841)), and constipation (OR 1.365 (1.000–1.862)) (Table 3). The AUC of the developed model was 0.712 (0.649–0.774). When using a risk threshold of 5%, the sensitivity and specificity of the developed model were 72% and 64%, respectively, whereas the positive and negative predictive values were 7% and 98%, respectively (Table 3).

**Table 3:** Predictor coefficients and model performance of the developed model for serious infections in patients with rheumatoid arthritis treated with tocilizumab

	Developed model (*N* = 5199) (non-zero predictors = 17)	
Predictors	Coefficient	Odds ratio (95% CI^b^)
Intercept	− 4.591	0.010 (0.005–0.020)
Sex (male)	0.062	1.064 (1.000–1.419)
Age, years	0.013	1.013 (1.002–1.024)
History of infections, ever (yes)	0.152	1.164 (1.000–1.924)
History of serious infections within 1 year (yes)	0.943	2.569 (1.636–3.745)
Constipation, ever (yes)	0.311	1.365 (1.000–1.862)
Renal disease, ever (yes)	0.031	1.031 (1.000–1.450)
Heart disease, ever (yes)	0.330	1.391 (1.006–1.841)
Vascular disease, ever (yes)	0.194	1.214 (1.000–1.617)
Diverticulitis and related disorders, ever (yes)	0.781	2.183 (1.000–3.989)
Amyloidosis, ever (yes)	0.053	1.055 (0.886–2.190)
Gastrointestinal ulcers, ever (yes)	0.197	1.218 (1.000–1.548)
Knee or hip prostheses within 5 years (yes)	0.130	1.138 (1.000–2.001)
Diabetes, ever (yes)	0.061	1.063 (1.000–1.392)
Chronic kidney diseases within 5 years (yes)	0.084	1.087 (1.000–1.589)
Malignancies within 10 years (yes)^a^	0.203	1.225 (1.000–1.643)
Steroid use within 1 year (yes)	0.151	1.163 (1.000–1.638)
MTX use within 1 week (yes)	− 0.191	0.826 (0.635–1.000)
**Model performance**		
Discrimination		
Cross-entropy (cross-validation in the training set)	0.1820	
Cross-entropy (test set)	0.1488	
AUC (test set)^b^	0.712	
Confidence interval AUC 95% (test set)^c^	0.649–0.774	
Accuracy at a threshold of 5% predicted risk in the test set		
Sensitivity	33/46 (72%)	
Specificity	802/1256 (64%)	
Positive predictive value	33/487 (7%)	
Negative predictive value	802/815 (98%)	

*Abbreviations* *AUC* area under the receiver operating characteristic curve, *CI* confidence interval, *MTX* methotrexate; The developed model was selected for the lowest average cross-validated cross-entropy in the training set, which includes a subset of the full analysis set (80% of the overall population) to train the prediction model. The test set comprised the latest 20% of eligible patients of the full analysis dataset, which is considered a temporal validation of the developed prediction model

^a^Only patients without active malignancy treatment at the index date

^b^95 CI obtained using DeLong’s method implemented in R package pROC. DeLong’s method from: [39] DeLong ER, DeLong DM, Clarke-Pearson DL. Comparing the areas under two or more correlated receiver operating characteristic curves: a nonparametric approach. Biometrics. 1988 Sep;44(3):837–45

^c^95% CI obtained using the bootstrap method with 1000 replicates

#### Comparison among the prediction models

The developed model was compared with the existing DANBIO and PMS models. The predictors included in the DANBIO model included age (categorised) and the count of risk factors (including previous SIs within 5 years, respiratory disease, diabetes, myocardial infarction, inflammatory bowel disease (IBD), and steroid use). The predictors included in the PMS model were age (≥ 65 years), duration of RA (≥ 10 years), respiratory diseases, and steroid use (> 0 to ≤ 5 mg/day, > 5 mg/day) (Table 4).

**Table 4:** Predictor coefficients and performance of the developed prediction model, DANBIO model, and PMS model

	Developed model	DANBIO model	PMS model
Predictors	Coefficient	Coefficient	Coefficient
Intercept	− 4.591	− 3.890	− 4.605
Sex (male)	0.062		
Age, years	0.013		
Age, categorized (40–49 years)		0.134	
(50–59 years)		0.423	
(60–69 years)		0.690	
(≥ 70 years)		1.231	
(≥ 65 years)			0.432
Duration of RA (≥ 10 years			0.582
History of infections, ever	0.152		
History of serious infections within 1 year	0.943		
Constipation, ever	0.311		
Renal diseases, ever	0.031		
Respiratory diseases, ever			0.658
Heart diseases, ever	0.330		
Vascular diseases, ever	0.194		
Diverticulitis, ever	0.781		
Amyloidosis, ever	0.053		
Gastrointestinal ulcers, ever	0.197		
Knee or hip prostheses within 5 years	0.130		
Diabetes, ever	0.061		
Chronic kidney diseases within 5 years	0.084		
Malignancies within 10 years^a^	0.203		
Steroid use within 1 year	0.151		
Steroids, categorized(> 0 to ≤ 5 mg/day)			− 0.041
(> 5 mg/day)			1.026
MTX use, within 1 week	− 0.191		
Count of 6 risk factors(1^b^)		0.280	
(≥ 2^b^)		1.212	
**Model performance**			
Discrimination			
Cross-entropy (cross-validation in the training set)	0.1820		
Cross-entropy (test set)^c^	0.1488	0.1932	0.1561
AUC (test set)^c^	0.712	0.591	0.565
Accuracy at a threshold of 5% predicted risk in the test set			
Sensitivity	33/46 (72%)	44/46 (96%)	9/46 (20%)
Specificity	802/1256 (64%)	233/1256 (19%)	1028/1256 (82%)
Positive predictive value	33/487 (7%)	44/1067 (4%)	9/237 (4%)
Negative predictive value	802/815 (98%)	233/235 (99%)	1028/1065 (97%)

*Abbreviations* *AUC* area under the receiver operating characteristic curve, *MTX* methotrexate, *PMS* postmarketing surveillance; The developed model was selected for the lowest average cross-validated cross-entropy in the training set, a subset of the full analysis set (80% of the overall population) to train the predictive models. DANBIO model from: Krabbe S, Grøn KL, Glintborg B, Nørgaard M, Mehnert F, Jarbøl DE, et al. Risk of serious infections in arthritis patients treated with biological drugs: a matched cohort study and development of prediction model. Rheumatology (Oxford). 2021 Aug 2;60(8):3834–44. PMS (postmarketing surveillance) model from Koike T, Harigai M, Inokuma S, Ishiguro N, Ryu J, Takeuchi T, et al. Effectiveness and safety of tocilizumab: postmarketing surveillance of 7901 patients with rheumatoid arthritis in Japan. J Rheumatol. 2014 Jan;41(1):15–23

^a^Only patients without the active malignancy treatment at the index date

^b^Count of six risk factors: a history of serious infection within 5 years, respiratory disease, diabetes, myocardial infarction, inflammatory bowel disease, and steroid use

^c^Estimated in the test set comprising the latest 20% of eligible patients of the full analysis dataset, which is considered a temporal validation of the developed prediction model. For DANBIO and PMS models, this is considered an external validation

The developed model had the lowest cross-entropy value (0.1488) compared to the values for DANBIO (0.1932) or PMS (0.1561) models (assessed in the test data set). The developed model had a higher AUC (0.712) compared to DANBIO (0.591) or PMS (0.565) models. Further information regarding the model discrimination is presented in Table 4.

**Fig. 1:**
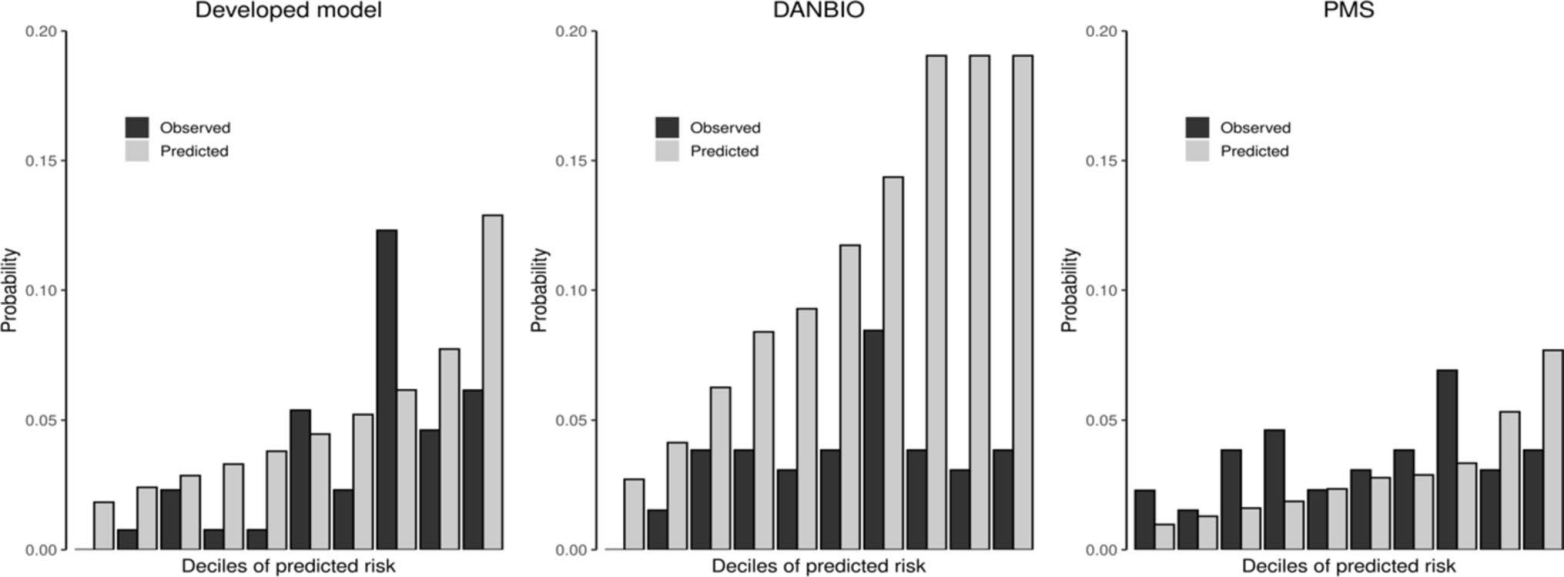
Calibration of the developed model, DANBIO model, and PMS model. Notes: the developed model was selected for the lowest average cross-validated cross-entropy in the training set, a subset of the full analysis set (80% of the overall population) to train the predictive models. DANBIO from: Krabbe S, Grøn KL, Glintborg B, Nørgaard M, Mehnert F, Jarbøl DE, et al. Risk of serious infections in arthritis patients treated with biological drugs: a matched cohort study and development of prediction model. Rheumatology (Oxford). 2021 Aug 2;60(8):3834–44. PMS (postmarketing surveillance) model from: Koike T, Harigai M, Inokuma S, Ishiguro N, Ryu J, Takeuchi T, et al. Effectiveness and safety of tocilizumab: postmarketing surveillance of 7901 patients with rheumatoid arthritis in Japan. J Rheumatol. 2014 Jan;41(1):15–23

The agreement between the observed and expected risk of SI varied between the models (Fig. 1). The DANBIO model tended to overestimate the risk of SI, whereas the PMS model tended to underestimate the risk of SI (except at highest predicted risk deciles). In the developed model, the agreement between the observed and expected probabilities varied by the predicted risk level.

**Fig. 2:**
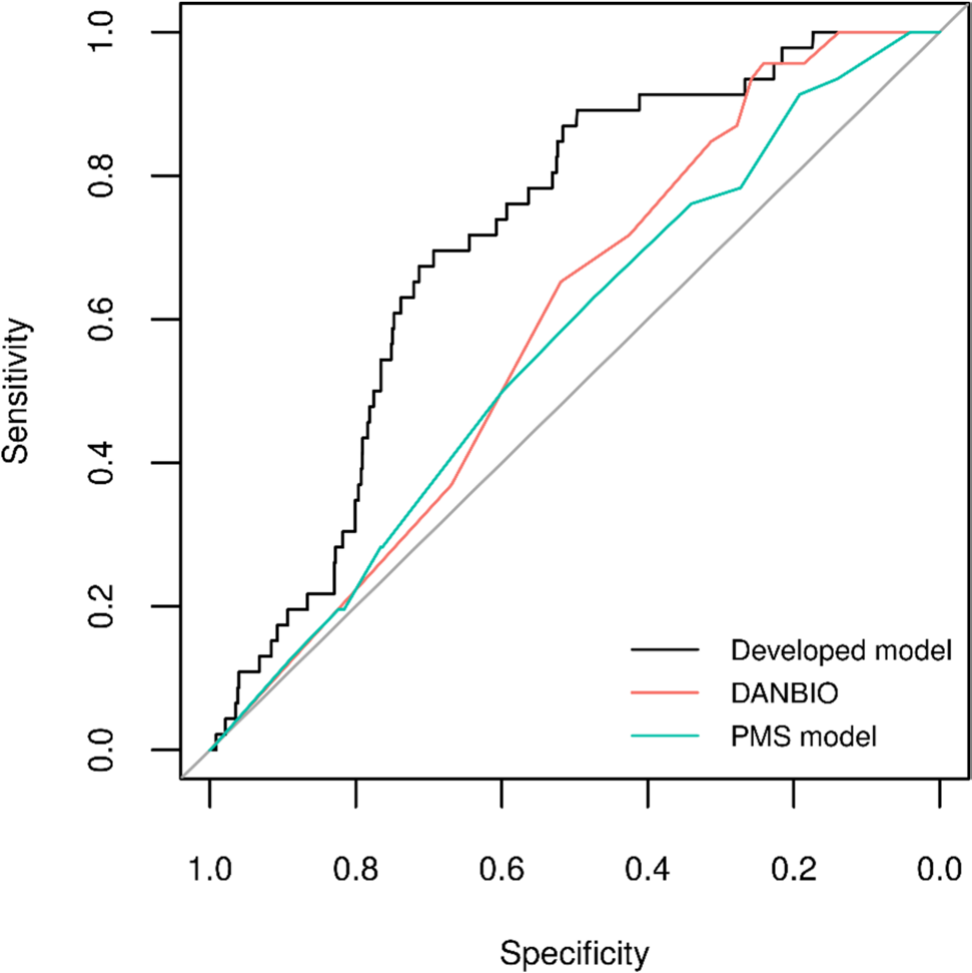
Receiver operating characteristic (ROC) curves of the developed model, DANBIO model, and PMS model. ROC: receiver operating characteristic curve. Notes: the developed model was selected for the lowest average cross-validated cross-entropy in the training set, a subset of the full analysis set (80% of the overall population) to train the predictive models. DANBIO model from: Krabbe S, Grøn KL, Glintborg B, Nørgaard M, Mehnert F, Jarbøl DE, et al. Risk of serious infections in arthritis patients treated with biological drugs: a matched cohort study and development of prediction model. Rheumatology (Oxford). 2021 Aug 2;60(8):3834–44. PMS (postmarketing surveillance) model from Koike T, Harigai M, Inokuma S, Ishiguro N, Ryu J, Takeuchi T, et al. Effectiveness and safety of tocilizumab: postmarketing surveillance of 7901 patients with rheumatoid arthritis in Japan. J Rheumatol. 2014 Jan;41(1):15–23

Figure 2 suggested that based on graphical comparison of corresponding ROC curves, among the models considered, the developed model had better overall ability to discriminate between patients experiencing SI event and event-free patients.

## Discussion

This study developed a risk prediction model for SI in RA patients treated with tocilizumab in Japan and evaluated the model’s performance. Of the 6501 patients, 4.57% experienced SIs during the 1-year follow-up period. The most frequent SIs were influenza and pneumonia, skin and subcutaneous tissue infections, and other bacterial diseases. The observed occurrence of SIs was consistent with those reported previously [23], although the differences in the SI definition, treatment, and patient characteristics (e.g., disease status and progression, functional impairment, and medical history) should be considered [33].

Of the ten developed candidate prediction models, the model with the lowest average cross-validated cross-entropy was preferred (referred to as the developed model). The developed model included 17 predictors of SI, such as demographics, a history of infections/SIs, concomitant disease (constipation), comorbidities (e.g., diverticulitis and heart diseases), malignancies, and co-treatment. The predictors showing the strongest associations with SI were a history of previous SI (within 1 year), consistent with previous studies [23, 25], and having diverticulitis (ever) as a comorbidity, which was not included in DANBIO [23] or PMS [25] models. In this study, a small number of patients (1.2%) had a history of diverticulitis; however, these patients may have a higher likelihood of re-hospitalization. Diverticulitis was a part of the outcome definition and may be reflected in the observed high coefficient compared to other predictors. However, future studies should confirm whether diverticulitis represents a risk factor for SI in RA patients treated with tocilizumab.

Of the other predictors, age showed a weak positive association with SI. The results regarding steroid use (within 1 year, indicating long-term use) showed a positive association with SI risk. The results are in line with the observations made in the DANBIO model [23] and in other studies assessing SI risk in RA patients [21, 34, 35]. MTX use (within 1 week), on the other hand, was negatively associated with SI risk. This may be due to patients who cannot use MTX (i.e., complications or other reasons). Respiratory disease is a known risk factor for infections; however, it is only included in Model 5, where it showed a negative association. This may be caused by an association between respiratory disease and infection, and variables of histories of infection may represent them in the shrinkage model. Therefore, this finding does not rule out respiratory disease as a risk factor for SI. These results should be interpreted carefully considering the uncertainty of estimation represented by 95% CIs. In addition, this study aimed to develop and evaluate prediction models, not to infer an etiology of SIs in the RA populations. There is a limitation in interpreting a predictor as a causal [36].

In terms of model performance, the developed model showed the lowest cross-entropy value compared with DANBIO [23] and PMS [25] models. Additionally, the developed model had a higher AUC compared to DANBIO [23] and PMS [25] models, indicating that the developed model had a better predictive ability to discriminate patients experiencing SIs from event-free patients.

The strength of this study is the notable sample of RA patients treated with tocilizumab in Japan. Furthermore, 27 potential predictors of SI, including known risk factors and potential predictors, and their variations were considered as candidate predictors. A strength is also the use of a validated definition of the outcome [30] and the extensive external validation to understand the prediction performance of the developed model.

The following limitations warrant consideration when interpreting the findings. The patient selection did not comply with the 2010 American College of Rheumatology/European League Against Rheumatism classification criteria for rheumatoid arthritis [37] since the database did not have detailed information for RA classification, and some patients were diagnosed before the publication of the latest classification. To mitigate the risk of inclusion of patients using tocilizumab for diseases other than RA, specific inclusion (i.e., definite RA diagnosis, a history of ≥ 2 antirheumatic drug prescriptions) and exclusion (i.e., tocilizumab prescribed for disease other than RA) criteria were used. Nevertheless, a 6-month baseline period before the first tocilizumab initiation may be relatively short to capture diagnostic information, thus excluding some RA patients. However, any bias stemming from this is expected to be low as diagnostic information for the drug prescription needed for reimbursement is typically continuously recorded in the clinical practice in Japan. Furthermore, the potential risk of selection bias stemming from the exclusion criteria of no prescription of tocilizumab after the index date (e.g., excluding severe patients who did not get the second prescription due to adverse events) should be acknowledged. Considering the administration of tocilizumab for RA (i.e., every 2–4 weeks) [14, 15], it is expected that the initial tocilizumab prescription is followed by subsequent prescription, thus reducing the bias.

The MDV database included data from acute hospitals in Japan; therefore, the study population may include more severe RA patients, potentially reflected as differences in the study population characteristics (e.g., RA disease severity and medical histories) compared to other studies. These characteristics of the study population may limit the generalizability of the results for all RA patients or patients in other geographical locations.

Limitations related to the development of the prediction model should be acknowledged. The predictors of the DANBIO [23] or PMS [25] models were defined according to operational definitions feasible in this data set; therefore, the information captured in these variables might differ from that captured in datasets used to develop these models. Consequently, the predictive effects of variables may be different between the datasets and impact the predictive performance. Also, the differences in the study population (e.g., patient demographics, RA disease severity, and medical histories) should be considered when comparing the performance of the models. The comparison of the models and the interpretation of the results should also consider the differences in the follow-up period, with the PMS model having 28 weeks [25] while in the developed and DANBIO [23] models having 1 year.

Moreover, the secondary use of administrative claims data for research purposes limits the availability and accuracy of all variables potentially relevant for a prediction model (e.g., variables unavailable or recorded inconsistently) or variables that could be important predictors for SI (e.g., RA disease activity) [22]. As a result, the lack of RA disease activity level as a predictor may result in biased estimates of the coefficient of other variables, especially those associated with RA disease activity (e.g., the use of MTX or steroids), and the performance and calibration of the developed model are limited to the inherent predictive ability of the available variables.

It should also be considered that the data-driven approaches for model selection can lead to overfitting and do not ensure the model selection that generalises best to new patients. To mitigate this issue, cross-validation methods were used when selecting the best models among all candidates.

Despite the limitations, this model is of great clinical importance to predict SIs in this population, as RA patients treated with tocilizumab may be at risk of SIs [18]. The developed model, along with identifying the main risk factors for SIs, may inform physicians on the SIs risk in RA patients in Japan and can contribute to personalised decision-making, early identification of patients with high-risk profiles to SIs, and reduction in associated morbidity, mortality, and costs. However, the developed model is based on secondary data; thus, the evidence is limited to recommend for its use in clinical practice. The implementation of this model in clinical practice should consider that population and measurement of predictors and outcomes vary and change over time [38]. Future research is needed to assess the feasibility of implementing this model in clinical practice and the implementation of such a model in software to automatically identify patients at high risk of SIs.

## Supplementary Information

Below is the link to the electronic supplementary material.

ESM 1 (DOCX 133 KB)

ESM 2 (XLS 94.5 KB)

## Data Availability

The data underlying this article were provided by Medical Data Vision Co., Ltd. under licence. Data will be shared upon a reasonable request to the corresponding author with the permission of Medical Data Vision Co., Ltd. The registration information for this study is available at the UMIN Clinical Trials Registry (UMIN000050079). The study protocol (in Japanese) will be shared upon reasonable request to the corresponding author, but with regret, analysis codes will not be available. The study protocol was prepared without patients or public involvement.
