# Supplementary material for: Development and validation of a prediction model for serious infections in rheumatoid arthritis patients treated with tocilizumab in Japan
Source: Clin Rheumatol. 2025 Feb 7;44(3):1081–93. doi: 10.1007/s10067-025-07328-9 (PMC11865113; doi:10.1007/s10067-025-07328-9)
Supplement: Supplementary file 1 — (DOCX 133 KB) [file 10067_2025_7328_MOESM1_ESM.docx]

ORIGINAL RESEARCH

Toshihiro Nanki et al

**Development and validation of a prediction model for serious infections in rheumatoid arthritis patients treated with tocilizumab in Japan**

Toshihiro Nanki^1*^, Tomohiro Yamaguchi^2^, Kosei Umetsu^2^, Ryunosuke Tanabe^2^, Naoki Maeda^2^, Minori Kanazawa^2^, Yuko Furuno^2^, Shinichi Matsuda^2^, Shinya Takemoto^2^, Keiko Asao^3^, Tatsuya Kamiuchi^2^

^1^Division of Rheumatology, Department of Internal Medicine, Toho University School of Medicine, Tokyo, Japan. ^2^Drug Safety Division Chugai Pharmaceutical Co. Ltd, Tokyo, Japan. ^3^IQVIA Solutions Japan G.K, Tokyo, Japan.

^*^Correspondence to: Toshihiro Nanki, 6-11-1 Omorinishi, Ota-ku, Tokyo 143-8541, Japan. Tel +81-3-3762-4151 Fax +81-3-5767-7292. Email: toshihiro.nanki@med.toho-u.ac.jp

# **Supplementary Table S1.** Inclusion and exclusion criteria applied in this study, their definitions and time of assessments

|  | **Criteria** | **Definition and time of assessment** | | |  |
| --- | --- | --- | --- | --- | --- |
| **Inclusion criteria** | Diagnosis of rheumatoid arthritis | Patients with a definite diagnosis of RA (excluding diagnoses with “suspected flag”^a^) during the baseline period. RA diagnosis identified by ICD-10 codes M05 or M06. | | |  |
|  | Age ≥18 years | Patients aged 18 years or more at the index date. | | |  |
|  | Prescription of tocilizumab | Prescription of tocilizumab during the patient selection period. Tocilizumab prescription identified by Japanese receipt code as described below, administrated either as intravenous or subcutaneous treatment. | | |  |
|  |  | **Drug** | **Generic name** | **Japanese receipt code** |  |
|  |  | Tocilizumab | Tocilizumab | 620006184, 620006804, 620006805, 622242201, 622242301 |  |
|  | History of two or more records of antirheumatic drugs | History of two or more records of antirheumatic drug prescriptions during the baseline period. Considered antirheumatic drugs as described below. Identified by Japanese receipt code. | | |  |
|  |  | **Drug** | **Generic name** | **Japanese receipt code** |  |
|  |  | MTX | Methotrexate | 610432016, 614210098, 620004082, 620004083, 620004084, 620007515, 621622602, 621642203, 621734801, 622221301, 622581501, 622630501, 622634501, 622642401, 622742200, 622742300, 622841001, 622869601, 622928001, 622928101, 622928201, 622928301, 644210048, 644210049, 620002151, 620004005, 620006686 |  |
|  |  | csDMARD | Bucillamine | 620000180, 620000181, 620006092, 620008052, 620942203, 620942401, 620943101, 622108402, 622743000, 622743100, 610422247, 610433106, 610433107, 610433151, 610433156, 622108401 |  |
|  |  |  | Salazosulfapyridine | 610463004, 620004897, 620008624, 620009119, 621133002, 621442802, 621442905, 621443002, 621443003, 622023001, 622039002, 622161402, 622173801, 622173901, 622746800, 622746900, 622747000, 622833801, 622833802, 616210018, 620006949, 610453008, 610453051, 610453065, 621443001, 622103601, 622103602, 622161401 |  |
|  |  |  | Tacrolimus hydrate | 610409342, 610443059, 610451009, 610451010, 613990096, 620008437, 620008438, 620008439, 622047401, 622232201, 622232202, 622232301, 622232303, 622232401, 622232403, 622270501, 622270601, 622270701, 622280901, 622281001, 622281101, 622281201, 622281301, 622370001, 622370101, 622370201, 622384303, 622384403, 622384503, 622384603, 622384703, 622437901, 622438001, 622438101, 622438601, 622438701, 622438801, 622580901, 622590901, 643990141, 622384301, 622384401, 622384501, 622384601 |  |
|  |  |  | Iguratimod | 622184501, 622882301, 622921701, 622929701, 622183501 |  |
|  |  |  | Leflunomide | 620000416, 620000417, 620000418 |  |
|  |  | Gold preparations | Sodium aurothiomalate | 640453080, 640453081 |  |
|  |  | TNFi | Infliximab | 640462006, 622378801, 622577001, 622590801, 622596901, 622651501 |  |
|  |  |  | Etanercept | 620002479, 620007231, 621934101, 622003901, 622242701, 622624701, 622634801, 622634901, 622635001, 622635101, 622635201, 622676001, 622676101, 622676201, 622676301, 629900201, 629900301, 629900401, 629900501, 629906301, 629918301, 629918401 |  |
|  |  |  | Adalimumab | 622509701, 622509801, 622608801, 622608901, 622609001, 629911201, 629911301, 629911401, 629913501, 629913601, 629913701, 629917401, 629917501, 629917601, 629917701, 620006808, 622093601 |  |
|  |  |  | Golimumab | 622070002, 622675801 |  |
|  |  |  | Certolizumab | 622224501, 622654701 |  |
|  |  | IL-6R Ab | Sarilumab | 622596601, 622596701, 622662001, 622662101 |  |
|  |  | T-cell activation | Abatacept | 622002901, 622265701, 622491601 |  |
|  |  | JAK inhibitors | Tofacitinib citrate | 622242601 |  |
|  |  |  | Baricitinib | 622573701, 622573801 |  |
|  |  |  | Peficitinib hydrobromide | 622678101, 622678201 |  |
|  |  |  | Upadacitinib hydrate | 622793801, 622793901, 622875101, 622920101 |  |
|  |  |  | Filgotinib maleate | 622830601, 622830701 |  |
|  |  |  |  |  |  |
|  |  | Glucocorticoids | Betamethasone | 620002613, 620002614, 620002615, 620002616, 620002617, 620003829, 620003830, 620003831, 620006275, 620006276, 620008220, 620004578, 620004620, 620005133, 620005134, 620006161, 620006162, 642450087 |  |
|  |  |  | Cortisone | 620002513 |  |
|  |  |  | Dexamethasone | 620003832, 620007336, 620525001, 620525101, 620525201, 620525301, 620525401, 620525601, 620525701, 620525801, 620525901, 640422045, 640422046, 640422047, 642450007, 642450008, 642450054, 642450055, 642450056, 612450017, 612450121, 620006985, 620006986, 620008651, 620521302, 621997701, 622359901 |  |
|  |  |  | Hydrocortisone | 620007332, 620007333, 620007334, 620007335, 620008816, 620008817, 620008818, 620008819, 620518605, 620518905, 620519005, 640454009, 642450029, 642450044, 642450046, 620002208, 620002209, 620005162, 620005163, 620519503, 620519603, 622756200, 612450007, 620006903 |  |
|  |  |  | Methylprednisolone | 620001310, 620003758, 620003759, 620007356, 620007357, 620007358, 620007359, 620528103, 620528502, 620528505, 620528901, 620528905, 620529204, 622100401, 622100501, 622329500, 622329600, 622329700, 622756500, 640454024, 640454025, 640454026, 642450049, 642450050, 642450051, 642450139, 642450140, 642450141, 642450142, 620007381, 620007382, 642450067, 620005125, 620005126 |  |
|  |  |  | Prednisolone | 620006613, 620006614, 620530402, 620530502, 642450115, 642450116, 642450117, 642450169, 642450170, 642450171, 610408661, 610422253, 610431117, 610454071, 612450051, 612450096, 612450118, 620000125, 620000694, 620000695, 620000696, 620000697, 620000698, 620001894, 620001895, 620001896, 620003272, 620004294, 620004387, 620005848, 620527133, 620527136, 620527205, 620527207, 621559301 |  |
|  |  |  | Triamcinolone | 620004660, 620004661, 612450070 |  |
| **Exclusion criteria** | No prescription of tocilizumab after the index date | Patients with no prescription of tocilizumab after the index date. Prescription of tocilizumab as described above. | | |  |
|  | A history of treatment for malignancies during the baseline period or on the index date | Patients with a record(s) of pharmacological treatment for malignancy (identified by ATC code L01 excluding L01BA and L01FA) and with a record(s) of malignancy (identified by ICD-10 codes C00-C97, D00-D09, D10-D19, D20-D29, D30-D36, D37-D48) during the baseline period or on the index date, or patients with a record(s) of radiologic treatment for malignancy (identified by Kubun codes M000, M000-21, M000-22, M000-23, M000-24, M000-25, M0001, M0002, M0003, M0004, M0005, M0006, M001-2, M001-31, M001-32, M001-33, M001-34, M001-41, M001-42, M001-43, M0012, M0013, M0015, M0016, M0017, M002, M0031, M0032, M0041, M0042, M0043, M0045, M0047) during the baseline period or on the index date. | | |  |
|  | No 4-month lookback period | Patients for whom data during the 4-month (120 days) lookback period preceding the index date cannot be ensured. | | |  |
|  | Prescription of tocilizumab for a disease other than RA during the baseline period or at the index date (except suspected disease). | Patients with tocilizumab prescription with a disease name other than RA for which tocilizumab was approved during the baseline period or on the index date. The considered diseases and corresponding ICD-10 code: juvenile idiopathic arthritis (M08), adult still disease (M06.1), Castleman’s disease (D47.7), pneumonia caused by SARS-CoV-2 (U07.1), cytokine release syndrome (T88.7), akayasu arteritis (M31.4), giant cell arteritis (M31.6). | | |  |

Abbreviations: ATC: Anatomical Therapeutic Chemical, csDMARD: conventional synthetic disease-modifying antirheumatic-rheumatic drug, ICD-10: International Classification of Diseases 10^th^ revision, IL6: interleukin-6, JAK: Janus kinase, MTX: Methotrexate, RA: Rheumatoid arthritis, SARS-CoV-2: severe acute respiratory syndrome coronavirus 2, TNFi: tumour necrosis factor.

Note: Baseline period is the 6-month (180 days) period preceding the index date. The index date is the first tocilizumab prescription during the patient selection period. The patient selection spans from 1^st^ April 2008 until 31^st^ July 2021.

^a^ A suspected flag refers to unconfirmed diagnosis.

# **Supplementary Table S2**. Infections considered in the definition of the serious infection outcome and the ICD-10 codes

| ICD-10 code | Disease name^a^ |
| --- | --- |
| A00-A09 | Intestinal infectious diseases |
| A15-A19 | Tuberculosis |
| A20-A28 | Certain zoonotic bacterial diseases |
| A30-A49 | Other bacterial diseases |
| A50-A60, A63, A64 | Infections with a predominantly sexual mode of transmission |
| A65-A69 | Other spirochaetal diseases |
| A70, A71, A74 | Other diseases caused by chlamydiae |
| A75, A77-A79 | Rickettsioses |
| A80-A89 | Viral infections of the central nervous system |
| A92-A99 | Arthropod-borne viral fevers and viral haemorrhagic fevers |
| B00-B09 | Viral infections characterised by skin and mucous membrane lesions |
| B15-B19 | Viral hepatitis |
| B20-B24- | Human immunodeficiency virus disease |
| B25-B27, B30, B33, B34 | Other viral diseases |
| B35-B49 | Mycoses |
| B50-B58, B60, B64 | Protozoal diseases |
| B65-B83 | Helminthiases |
| B85-B89 | Pediculosis, acariasis and other infestations |
| B90-B94 | Sequelae of infectious and parasitic diseases |
| B95-B98 | Bacterial, viral and other infectious agents |
| B99 | Other infectious diseases |
| G00 | Bacterial meningitis, not elsewhere classified |
| G03 | Meningitis due to other and unspecified causes |
| G04 | Encephalitis, myelitis and encephalomyelitis |
| G06 | Intracranial and intraspinal abscess and granuloma |
| G36 | Other acute disseminated demyelination |
| G37 | Other demyelinating diseases of central nervous system |
| G51 | Facial nerve disorders |
| G58 | Other mononeuropathies |
| G60 | Hereditary and idiopathic neuropathy |
| G62 | Other polyneuropathies |
| H00 | Hordeolum and chalazion |
| H01 | Other inflammation of eyelid |
| H04 | Disorders of lacrimal system |
| H05 | Disorders of orbit |
| H10 | Conjunctivitis |
| H16 | Keratitis |
| H20 | Iridocyclitis |
| H30 | Chorioretinal inflammation |
| H35 | Other retinal disorders |
| H44 | Disorders of globe |
| H46 | Optic neuritis |
| H60 | Otitis externa |
| H61 | Other disorders of external ear |
| H65 | Nonsuppurative otitis media |
| H66 | Suppurative and unspecified otitis media |
| H68 | Eustachian salpingitis and obstruction |
| H70 | Mastoiditis and related conditions |
| H73 | Other disorders of tympanic membrane |
| H81 | Disorders of vestibular function |
| H83 | Other diseases of inner ear |
| H93 | Other disorders of ear, not elsewhere classified |
| I00 | Rheumatic fever without mention of heart involvement |
| I01 | Rheumatic fever with heart involvement |
| I30 | Acute pericarditis |
| I31 | Other diseases of pericardium |
| I33 | Acute and subacute endocarditis |
| I38 | Endocarditis, valve unspecified |
| I40 | Acute myocarditis |
| I51 | Complications and ill-defined descriptions of heart disease |
| I71 | Aortic aneurysm and dissection |
| I72 | Other aneurysm and dissection |
| I74 | Arterial embolism and thrombosis |
| I77 | Other disorders of arteries and arterioles |
| I80 | Phlebitis and thrombophlebitis |
| I88 | Nonspecific lymphadenitis |
| I89 | Other noninfective disorders of lymphatic vessels and lymph nodes |
| J00-J06 | Acute upper respiratory infections |
| J09-J18 | Influenza and pneumonia |
| J20-J22 | Other acute lower respiratory infections |
| J31 | Chronic rhinitis, nasopharyngitis and pharyngitis |
| J32 | Chronic sinusitis |
| J34 | Other disorders of nose and nasal sinuses |
| J35 | Chronic diseases of tonsils and adenoids |
| J36 | Peritonsillar abscess |
| J37 | Chronic laryngitis and laryngotracheitis |
| J38 | Diseases of vocal cords and larynx, not elsewhere classified |
| J39 | Other diseases of upper respiratory tract |
| J40 | Bronchitis, not specified as acute or chronic |
| J41 | Simple and mucopurulent chronic bronchitis |
| J42 | Unspecified chronic bronchitis |
| J44 | Other chronic obstructive pulmonary disease |
| J85 | Abscess of lung and mediastinum |
| J86 | Pyothorax |
| J90 | Pleural effusion, not elsewhere classified |
| J98 | Other respiratory disorders |
| K05 | Gingivitis and periodontal diseases |
| K06 | Other disorders of gingiva and edentulous alveolar ridge |
| K07 | Dentofacial anomalies [including malocclusion] |
| K10 | Other diseases of jaws |
| K11 | Diseases of salivary glands |
| K12 | Stomatitis and related lesions |
| K13 | Other diseases of lip and oral mucosa |
| K14 | Diseases of tongue |
| K20 | Oesophagitis |
| K22 | Other diseases of oesophagus |
| K29 | Gastritis and duodenitis |
| K31 | Other diseases of stomach and duodenum |
| K35 | Acute appendicitis |
| K36 | Other appendicitis |
| K37 | Unspecified appendicitis |
| K52 | Other noninfective gastroenteritis and colitis |
| K57 | Diverticular disease of intestine |
| K61 | Abscess of anal and rectal regions |
| K62 | Other diseases of anus and rectum |
| K65 | Peritonitis |
| K75 | Other inflammatory liver diseases |
| K81 | Cholecystitis |
| K83 | Other diseases of biliary tract |
| K85 | Acute pancreatitis |
| K91 | Postprocedural disorders of digestive system, not elsewhere classified |
| L00-L05, L08 | Infections of the skin and subcutaneous tissue |
| L30 | Other dermatitis |
| L40 | Psoriasis |
| L66 | Cicatricial alopecia [scarring hair loss] |
| L73 | Other follicular disorders |
| L74 | Eccrine sweat disorders |
| L89 | Decubitus ulcer and pressure area |
| L98 | Other disorders of skin and subcutaneous tissue, not elsewhere classified |
| M00 | Pyogenic arthritis |
| M46 | Other inflammatory spondylopathies |
| M47 | Spondylosis |
| M60 | Myositis |
| M71 | Other bursopathies |
| M72 | Fibroblastic disorders |
| M86 | Osteomyelitis |
| N00 | Acute nephritic syndrome |
| N01 | Rapidly progressive nephritic syndrome |
| N10 | Acute tubule-interstitial nephritis |
| N05 | Unspecified nephritic syndrome |
| N12 | Tubulo-interstitial nephritis, not specified as acute or chronic |
| N15 | Other renal tubule-interstitial diseases |
| N28 | Other disorders of kidney and ureter, not elsewhere classified |
| N30 | Cystitis |
| N32 | Other disorders of bladder |
| N34 | Urethritis and urethral syndrome |
| N36 | Other disorders of urethra |
| N39 | Other disorders of urinary system |
| N41 | Inflammatory diseases of prostate |
| N45 | Orchitis and epididymitis |
| N48 | Other disorders of penis |
| N49 | Inflammatory disorders of male genital organs, not elsewhere classified |
| N50 | Other disorders of male genital organs |
| N61 | Inflammatory disorders of breast |
| N70 | Salpingitis and oophoritis |
| N71 | Inflammatory disease of uterus, except cervix |
| N72 | Inflammatory disease of cervix uteri |
| N73 | Other female pelvic inflammatory diseases |
| N75 | Diseases of Bartholin gland |
| N76 | Other inflammation of vagina and vulva |
| O23 | Infections of genitourinary tract in pregnancy |
| O35 | Maternal care for known or suspected fetal abnormality and damage |
| O41 | Other disorders of amniotic fluid and membranes |
| O85 | Puerperal sepsis |
| O86 | Other puerperal infections |
| O91 | Infections of breast associated with childbirth |
| O98 | Maternal infectious and parasitic diseases classifiable elsewhere but complicating pregnancy, childbirth and the puerperium |
| O99 | Other maternal diseases classifiable elsewhere but complicating pregnancy, childbirth and the puerperium |
| R02 | Gangrene, not elsewhere classified |
| R09 | Other symptoms and signs involving the circulatory and respiratory systems |
| T79 | Certain early complications of trauma, not elsewhere classified |
| T81 | Complications of procedures, not elsewhere classified |
| T82 | Complications of cardiac and vascular prosthetic devices, implants and grafts |
| T83 | Complications of genitourinary prosthetic devices, implants and grafts |
| T84 | Complications of internal orthopaedic prosthetic devices, implants and grafts |
| T85 | Complications of other internal prosthetic devices, implants and grafts |

Abbreviations: ICD-10: International Statistical Classification of Diseases and Related Health Problems, 10th revision.

^a^ The serious infections were defined as hospitalisation with the diagnosis of infection in the presence of immunological infection test and other related tests within 1 month before the date of hospitalisation or during the hospitalisation.

# **Supplementary Table S3.** Definitions of the baseline characteristics and predictors of serious infections, time of assessment and ascertainment

| **Domain** | **Variable** | **Time of assessment** | **Ascertainment** |
| --- | --- | --- | --- |
| **Demographics** | Sex (male/female)^a^ | Index date |  |
|  | Age (years) (mean, SD, median, Q1, Q3, min, max)^a^ | Index date |  |
|  | Age (<40, 40-49, 50-59, 60-69, ≥70) (years)^a^ | Index date |  |
| **RA disease characteristics** | Duration of RA (mean, SD, median, Q1, Q3, min, max) | The difference in years between the earliest RA diagnosis during the entire data period until the index date | RA diagnosis as described in Supplementary Table S1 |
|  | Duration of RA (<10 years, ≥10 years)^a^ | The difference in years between the earliest RA diagnosis during the entire data period until the index date | RA diagnosis as described in Supplementary Table S1 |
| **History of infections** | Infections (Yes/No)^a^ | The entire data period before the month of the index date | Infections, regardless of hospitalisation or immunological test, as described in Supplementary Table S2 |
|  | Serious infections, ever (Yes/No)^a^ | Entire data period before the month of  the index date* |  |
|  | Serious infections within 1 year (Yes/No)^a^ | 1 year period before the month of the index date* | Serious infection described in Supplementary Table S2 |
|  | Serious infections within 5 years (Yes/No) | 5 years period before the month of the index date* | Serious infection described in Supplementary Table S2 |
| **Concomitant disease** | Constipation (Yes/No)^a^ | The same month as the index date | ICD-10 code K59.0 |
| **Comorbidities** | Liver diseases, ever (Yes/No)^a^ | The entire data period until the index date | ICD-10 codes K70-K76 |
|  | Renal diseases, ever (Yes/No)^a^ | The entire data period until the index date | ICD-10 codes N00-N07, N10-N15, N17-N19 |
|  | Respiratory diseases, ever (Yes/No)^a^ | The entire data period until the index date | ICD-10 codes J30-J39, J40-J47, J60-J70, J80-J84, J85-J86, J90-J94, J95-J99 |
|  | Heart diseases, ever (Yes/No)^a^ | The entire data period until the index date | ICD-10 codes I01, I09, I11, I13, I20-25, I27, I30, I31, I33, I38, I40, I42, I44, I45-51 |
|  | Vascular diseases, ever (Yes/No)^a^ | The entire data period until the index date | ICD-10 codes I05-08, I10, I12, I15, I26, I28, I34-I38, I70-74, I77, I78, I80-83, I85-87 |
|  | Diverticulitis and related disorders (Yes/No)^a^ | The entire data period until the index date | ICD-10 code K57 |
|  | Amyloidosis (Yes/No)^a^ | The entire data period until the index date | ICD-10 code E85 |
|  | Gastrointestinal ulcers, ever (Yes/No)^a^ | The entire data period until the index date | ICD-10 codes K25-K28 |
|  | Knee or hip prostheses, ever (Yes/No)^a^ | The entire data period until the index date | Japanese receipt codes or Kubun (category) codes: Artificial femoral head insertion (hip), emergency insertion add-on fee (artificial he insertion), artificial joint reimplantation add-on fee (hip), artificial joint reimplantation add-on fee (knee), hip prosthesis sliding surface exchange, artificial joint replacement (hip), artificial joint replacement (knee). |
|  | Knee or hip prostheses within 5 years (Yes/No)^a^ | 5 years period prior to the index date | Kubun (category) codes: artificial femoral head insertion (hip), emergency insertion add-on fee (artificial head prosthesis insertion), artificial joint reimplantation add-on fee (hip), artificial joint reimplantation add-on fee (knee), hip prosthesis sliding surface exchange, artificial joint replacement (hip), artificial joint replacement (knee) |
|  | Interstitial lung diseases, ever (Yes/No)^a^ | The entire data period until the index date | ICD-10 codes J84, J70.2, J70.3, J70.4 |
|  | Interstitial lung diseases within 5 years (Yes/No)^a^ | 5 years period prior to the index date | ICD-10 codes J84, J70.2, J70.3, J70.4 |
|  | Diabetes, ever (Yes/No)^a^ | The entire data period until the index date | ICD-10 codes E10-E14 |
|  | Diabetes within 5 years (Yes/No)^a^ | 5 years period prior to the index date | ICD-10 codes E10-E14 |
|  | Myocardial infarction, ever (Yes/No)^a^ | The entire data period until the index date | ICD-10 codes I21-I23 |
|  | Myocardial infarction within 5 years (Yes/No)^a^ | 5 years period prior to the index date | ICD-10 codes I21-I23 |
|  | Chronic kidney disease, ever (Yes/No)^a^ | The entire data period until the index date | ICD-10 code N18 |
|  | Chronic kidney disease within 5 years (Yes/No)^a^ | 5 years period prior to the index date | ICD-10 code N18 |
|  | IBD, ever (Yes/No)^a^ | The entire data period until the index date | ICD-10 codes K50-K51 |
|  | IBD within 5 years (Yes/No)^a^ | 5 years period prior to the index date | ICD-10 codes K50-K51 |
|  | Malignancies (only the patients without the active malignancy treatment on the index date), ever (Yes/No) | The entire data period until the index date | Malignancies and corresponding ICD-10 code: Malignant neoplasm C00-C97, in situ neoplasms D00-D09, Benign neoplasms D10-D19, D20-D29, D30-D36, neoplasm of uncertain or unknown behaviour D37-D48  The pharmacological treatment for malignancy identified by ATC code L01 (excluding L01BA and L01FA)  Radiation therapy identified by Kubun codes M000, M000-21, M000-22, M000-23, M000-24, M000-25, M0001, M0002, M0003, M0004, M0005, M0006, M001-2, M001-31, M001-32, M001-33, M001-34, M001-41, M001-42, M001-43, M0012, M0013, M0015, M0016, M0017, M002, M0031, M0032, M0041, M0042, M0043, M0045, M0047 |
|  | Malignancies (only the patients without the active treatment on the index date) within 10 years (Yes/No)^a^ | 10 years period prior to the index date | Same as above. |
| **Laboratory data** | CRP (mg/dL) (mean, SD, median, Q1, Q3, min, max) | 2 weeks (14 days) period prior to the index date | As recorded in the data source |
| **Co-treatment** | DMARD use (Yes/No) | 1 week (7 days) period prior to the index date | Bucillamine, iguratimod, leflunomide, methotrexate, salazosulfapyridine, sodium aurothiomalate, tacrolimus, abatacept, adalimumab, certolizumab, etanercept, golimumab, infliximab, sarilumab, baricitinib, filgotinib, peficitinib, tofacitinib, and upadacitinib. Identified by using Japanese receipt codes |
|  | MTX use (Yes/No)^a^ | 1 week (7 days) period prior to the index date | Any methotrexate identified by Japanese receipt codes |
|  | Salazosulfrapyridine (Yes/No) | 1 week (7 days) period prior to the index date | Salazosulfrapyridine identified by Japanese receipt codes |
|  | Leflunomide (Yes/No) | 1 week (7 days) period prior to the index date | Leflunomide identified by Japanese receipt codes |
|  | TNF inhibitors/other biologic use (Yes/No) | 1 week (7 days) period prior to the index date | TNF inhibitors/other biologics identified by Japanese receipt codes |
|  | Oral steroid use (Yes/No) | 1 week (7 days) period prior to the index date | Cortisone, dexamethasone, triamcinolone, hydrocortisone, prednisolone, betamethasone, and methylprednisolone identified by Japanese receipt codes |
|  | Injection steroid use (Yes/No) | 1 week (7 days) period prior to the index date | Same as above, except for cortisone. |
|  | Any steroid use within 1 week (Yes/No)^a^ | 1 week (7 days) period prior to the index date | Same as above, including cortisone. |
|  | Any steroid use within 1 year (Yes/No)^a^ | 1 year period before the month of the  index date* | Same as above, including cortisone. |
|  | Steroid use (0, >0 to <=5, >5 mg/day) within 1 week (Yes/No)^a^ | 1 week (7 days) period prior to the index date | Same as above, including cortisone. |
| **History of RA treatment** | Number of previous treatments with non-biologic/biologic DMARDs (>5, ≤5) (Yes/No)^a^ | The entire data period before the index date* | As described above for DMARDs. |
|  | Number of previous treatments with csDMARDs (0, 1, 2, 3, ≥4) (Yes/No) | The entire data period before the index date* | Bucillamine, iguratimod, leflunomide, methotrexate, salazosulfapyridine, sodium aurothiomalate, tacrolimus. Identified by Japanese receipt codes. |

Abbreviations: ATC: Anatomical Therapeutic Chemical, CRP: C-creative protein, csDMARD: conventional synthetic disease-modifying antirheumatic-rheumatic drug, DMARD: disease-modifying antirheumatic drug, IBD: inflammatory bowel diseases, ICD-10: International Classification of Diseases, 10^th^ revision, Max: Maximum, MI: myocardial infarction, Min: Minimum, MTX: Methotrexate, Q1: first quartile, Q3: third quartile, RA: rheumatoid arthritis, SD: Standard deviation, TNF: Tumour Necrosis Factor.

Note: The index date is included in the time of assessment for the variables, except for the index date denoted with “*”, which indicates that the index date/index month is excluded. In case of multiple observations, the value closest to the index date was used (where applicable).

^a^ Indicates the predictors of serious infections

# **Supplementary Table S4.** Proportion of patients with rheumatoid arthritis prescribed with tocilizumab with serious infection, overall and by categories and subcategories

| ICD-10 | Overall population N=6501 | Training set  N=5199 | Test set N=1302 |
| --- | --- | --- | --- |
| Any serious infection, overall, n (%) | 297 (4.57%) | 251 (4.83%) | 46 (3.53%) |
| Serious infections by the subcategories, n (%) |  |  |  |
| A00-A09 Intestinal infectious diseases | 15 (0.23%) | 13 (0.25%) | 2 (0.15%) |
| A04.5 | 1 (6.67%) | 1 (7.69%) | 0 (0.00%) |
| A04.9 | 1 (6.67%) | 1 (7.69%) | 0 (0.00%) |
| A08.1 | 1 (6.67%) | 1 (7.69%) | 0 (0.00%) |
| A08.4 | 1 (6.67%) | 1 (7.69%) | 0 (0.00%) |
| A09.9 | 11 (73.33%) | 9 (69.23%) | 2 (100.00%) |
| A30-A49 Other bacterial diseases | 21 (0.32%) | 15 (0.29%) | 6 (0.46%) |
| A31.0 | 1 (4.76%) | 0 (0.00%) | 1 (16.67%) |
| A31.9 | 1 (4.76%) | 0 (0.00%) | 1 (16.67%) |
| A40.1 | 1 (4.76%) | 1 (6.67%) | 0 (0.00%) |
| A41.0 | 1 (4.76%) | 0 (0.00%) | 1 (16.67%) |
| A41.5 | 2 (9.52%) | 2 (13.33%) | 0 (0.00%) |
| A41.8 | 1 (4.76%) | 1 (6.67%) | 0 (0.00%) |
| A41.9 | 13 (61.90%) | 10 (66.67%) | 3 (50.00%) |
| A46 | 1 (4.76%) | 1 (6.67%) | 0 (0.00%) |
| A50-A64 Infections with a predominantly sexual mode of transmission | 1 (0.02%) | 1 (0.02%) | 0 (0.00%) |
| A63.0 | 1 (100.00%) | 1 (100.00%) | 0 (0.00%) |
| B00-B09 Viral infections characterised by skin and mucous membrane lesions | 16 (0.25%) | 15 (0.29%) | 1 (0.08%) |
| B00.4 | 1 (6.25%) | 1 (6.67%) | 0 (0.00%) |
| B02.1 | 2 (12.50%) | 2 (13.33%) | 0 (0.00%) |
| B02.7 | 1 (6.25%) | 1 (6.67%) | 0 (0.00%) |
| B02.9 | 12 (75.00%) | 11 (73.33%) | 1 (100.00%) |
| B15-B19 Viral hepatitis | 1 (0.02%) | 1 (0.02%) | 0 (0.00%) |
| B17.9 | 1 (100.00%) | 1 (100.00%) | 0 (0.00%) |
| B25-B34 Other viral diseases | 1 (0.02%) | 1 (0.02%) | 0 (0.00%) |
| B25.0 | 1 (100.00%) | 1 (100.00%) | 0 (0.00%) |
| B35-B49 Mycoses | 4 (0.06%) | 4 (0.08%) | 0 (0.00%) |
| B44.1 | 2 (50.00%) | 2 (50.00%) | 0 (0.00%) |
| B49 | 2 (50.00%) | 2 (50.00%) | 0 (0.00%) |
| G00-G09 Inflammatory diseases of the central nervous system | 2 (0.03%) | 2 (0.04%) | 0 (0.00%) |
| G03.0 | 1 (50.00%) | 1 (50.00%) | 0 (0.00%) |
| G04.9 | 1 (50.00%) | 1 (50.00%) | 0 (0.00%) |
| G50-G59 Nerve, nerve root and plexus disorders | 1 (0.02%) | 1 (0.02%) | 0 (0.00%) |
| G51.0 | 1 (100.00%) | 1 (100.00%) | 0 (0.00%) |
| G60-G64 Polyneuropathies and other disorders of the peripheral nervous system | 1 (0.02%) | 1 (0.02%) | 0 (0.00%) |
| G62.9 | 1 (100.00%) | 1 (100.00%) | 0 (0.00%) |
| H15-H22 Disorders of sclera, cornea, iris and ciliary body | 2 (0.03%) | 2 (0.04%) | 0 (0.00%) |
| H16.0 | 2 (100.00%) | 2 (100.00%) | 0 (0.00%) |
| H30-H36 Disorders of choroid and retina | 3 (0.05%) | 3 (0.06%) | 0 (0.00%) |
| H35.3 | 3 (100.00%) | 3 (100.00%) | 0 (0.00%) |
| H65-H75 Diseases of middle ear and mastoid | 1 (0.02%) | 1 (0.02%) | 0 (0.00%) |
| H66.0 | 1 (100.00%) | 1 (100.00%) | 0 (0.00%) |
| H80-H83 Diseases of inner ear | 5 (0.08%) | 5 (0.10%) | 0 (0.00%) |
| H81.1 | 2 (40.00%) | 2 (40.00%) | 0 (0.00%) |
| H81.3 | 1 (20.00%) | 1 (20.00%) | 0 (0.00%) |
| H81.9 | 2 (40.00%) | 2 (40.00%) | 0 (0.00%) |
| I30-I52 Other forms of heart disease | 4 (0.06%) | 3 (0.06%) | 1 (0.08%) |
| I31.8 | 1 (25.00%) | 0 (0.00%) | 1 (100.00%) |
| I31.9 | 2 (50.00%) | 2 (66.67%) | 0 (0.00%) |
| I33.0 | 1 (25.00%) | 1 (33.33%) | 0 (0.00%) |
| I70-I79Diseases of arteries, arterioles and capillaries | 7 (0.11%) | 6 (0.12%) | 1 (0.08%) |
| I71.0 | 4 (57.14%) | 4 (66.67%) | 0 (0.00%) |
| I72.3 | 1 (14.29%) | 1 (16.67%) | 0 (0.00%) |
| I74.3 | 2 (28.57%) | 1 (16.67%) | 1 (100.00%) |
| I80-I89 Diseases of veins, lymphatic vessels and lymph nodes, not elsewhere classified | 3 (0.05%) | 1 (0.02%) | 2 (0.15%) |
| I80.2 | 3 (100.00%) | 1 (100.00%) | 2 (100.00%) |
| J09-J18 Influenza and pneumonia | 54 (0.83%) | 45 (0.87%) | 9 (0.69%) |
| J10.1 | 1 (1.85%) | 1 (2.22%) | 0 (0.00%) |
| J13 | 4 (7.41%) | 3 (6.67%) | 1 (11.11%) |
| J14 | 1 (1.85%) | 1 (2.22%) | 0 (0.00%) |
| J15.0 | 1 (1.85%) | 1 (2.22%) | 0 (0.00%) |
| J15.7 | 1 (1.85%) | 1 (2.22%) | 0 (0.00%) |
| J15.9 | 14 (25.93%) | 12 (26.67%) | 2 (22.22%) |
| J18.0 | 4 (7.41%) | 4 (8.89%) | 0 (0.00%) |
| J18.1 | 4 (7.41%) | 3 (6.67%) | 1 (11.11%) |
| J18.8 | 1 (1.85%) | 0 (0.00%) | 1 (11.11%) |
| J18.9 | 23 (42.59%) | 19 (42.22%) | 4 (44.44%) |
| J20-J22 Other acute lower respiratory infections | 2 (0.03%) | 2 (0.04%) | 0 (0.00%) |
| J20.9 | 1 (50.00%) | 1 (50.00%) | 0 (0.00%) |
| J21.9 | 1 (50.00%) | 1 (50.00%) | 0 (0.00%) |
| J30-J39 Other diseases of upper respiratory tract | 3 (0.05%) | 2 (0.04%) | 1 (0.08%) |
| J32.4 | 1 (33.33%) | 1 (50.00%) | 0 (0.00%) |
| J32.9 | 1 (33.33%) | 1 (50.00%) | 0 (0.00%) |
| J36 | 1 (33.33%) | 0 (0.00%) | 1 (100.00%) |
| J40-J47 Chronic lower respiratory diseases | 1 (0.02%) | 1 (0.02%) | 0 (0.00%) |
| J42 | 1 (100.00%) | 1 (100.00%) | 0 (0.0%) |
| J85-J86 Suppurative and necrotic conditions of lower respiratory tract | 2 (0.03%) | 1 (0.02%) | 1 (0.08%) |
| J85.2 | 1 (50.00%) | 0 (0.00%) | 1 (100.00%) |
| J86.9 | 1 (50.00%) | 1 (100.00%) | 0 (0.00%) |
| J90-J94 Other diseases of pleura | 2 (0.03%) | 2 (0.04%) | 0 (0.00%) |
| J90 | 2 (100.00%) | 2 (100.00%) | 0 (0.00%) |
| J95-J99 Other diseases of the respiratory system | 1 (0.02%) | 1 (0.02%) | 0 (0.00%) |
| J98.2 | 1 (100.00%) | 1 (100.00%) | 0 (0.00%) |
| K20-K31 Diseases of oesophagus, stomach and duodenum | 3 (0.05%) | 3 (0.06%) | 0 (0.00%) |
| K29.5 | 1 (33.33%) | 1 (33.33%) | 0 (0.00%) |
| K31.5 | 1 (33.33%) | 1 (33.33%) | 0 (0.00%) |
| K31.8 | 1 (33.33%) | 1 (33.33%) | 0 (0.00%) |
| K35-K38 Diseases of appendix | 4 (0.06%) | 4 (0.08%) | 0 (0.00%) |
| K35.3 | 2 (50.00%) | 2 (50.00%) | 0 (0.00%) |
| K35.8 | 2 (50.00%) | 2 (50.00%) | 0 (0.00%) |
| K50-K52 Noninfective enteritis and colitis | 1 (0.02%) | 1 (0.02%) | 0 (0.00%) |
| K52.1 | 1 (100.00%) | 1 (100.00%) | 0 (0.00%) |
| K55-K64 Other diseases of intestines | 12 (0.18%) | 12 (0.23%) | 0 (0.00%) |
| K57.0 | 1 (8.33%) | 1 (8.33%) | 0 (0.00%) |
| K57.3 | 10 (83.33%) | 10 (83.33%) | 0 (0.00%) |
| K61.0 | 1 (8.33%) | 1 (8.33%) | 0 (0.00%) |
| K65-K67 Diseases of peritoneum | 6 (0.09%) | 5 (0.10%) | 1 (0.08%) |
| K65.0 | 6 (100.00%) | 5 (100.00%) | 1 (100.00%) |
| K70-K77 Diseases of liver | 1 (0.02%) | 1 (0.02%) | 0 (0.00%) |
| K75.0 | 1 (100.00%) | 1 (100.00%) | 0 (0.00%) |
| K80-K87 Disorders of gallbladder, biliary tract and pancreas | 13 (0.20%) | 13 (0.25%) | 0 (0.00%) |
| K81.0 | 1 (7.69%) | 1 (7.69%) | 0 (0.00%) |
| K83.0 | 3 (23.08%) | 3 (23.08%) | 0 (0.00%) |
| K83.1 | 1 (7.69%) | 1 (7.69%) | 0 (0.00%) |
| K83.3 | 1 (7.69%) | 1 (7.69%) | 0 (0.00%) |
| K85.0 | 1 (7.69%) | 1 (7.69%) | 0 (0.00%) |
| K85.1 | 1 (7.69%) | 1 (7.69%) | 0 (0.00%) |
| K85.9 | 5 (38.46%) | 5 (38.46%) | 0 (0.00%) |
| K90-K93 Other diseases of the digestive system | 3 (0.05%) | 3 (0.06%) | 0 (0.00%) |
| K91.3 | 3 (100.00%) | 3 (100.00%) | 0 (0.00%) |
| L00-L08 Infections of the skin and subcutaneous tissue | 38 (0.58%) | 28 (0.54%) | 10 (0.77%) |
| L02.0 | 1 (2.63%) | 1 (3.57%) | 0 (0.00%) |
| L02.1 | 1 (2.63%) | 1 (3.57%) | 0 (0.00%) |
| L02.3 | 2 (5.26%) | 2 (7.14%) | 0 (0.00%) |
| L02.4 | 4 (10.53%) | 3 (10.71%) | 1 (10.00%) |
| L03.1 | 23 (60.53%) | 15 (53.57%) | 8 (80.00%) |
| L03.2 | 1 (2.63%) | 1 (3.57%) | 0 (0.00%) |
| L03.9 | 5 (13.16%) | 4 (14.29%) | 1 (10.00%) |
| L04.9 | 1 (2.63%) | 1 (3.57%) | 0 (0.00%) |
| L40-L45 Papulosquamous disorders | 1 (0.02%) | 1 (0.02%) | 0 (0.00%) |
| L40.1 | 1 (100.00%) | 1 (100.00%) | 0 (0.00%) |
| L80-L99 Other disorders of the skin and subcutaneous tissue | 3 (0.05%) | 3 (0.06%) | 0 (0.00%) |
| L98.4 | 3 (100.00%) | 3 (100.00%) | 0 (0.00%) |
| M00-M25 Arthropathies | 11 (0.17%) | 10 (0.19%) | 1 (0.08%) |
| M00.01 | 1 (9.09%) | 1 (10.00%) | 0 (0.00%) |
| M00.92 | 1 (9.09%) | 1 (10.00%) | 0 (0.00%) |
| M00.93 | 2 (18.18%) | 2 (20.00%) | 0 (0.00%) |
| M00.94 | 3 (27.27%) | 2 (20.00%) | 1 (100.00%) |
| M00.95 | 2 (18.18%) | 2 (20.00%) | 0 (0.00%) |
| M00.97 | 2 (18.18%) | 2 (20.00%) | 0 (0.00%) |
| M40-M54 Dorsopathies | 14 (0.22%) | 10 (0.19%) | 4 (0.31%) |
| M46.26 | 1 (7.14%) | 1 (10.00%) | 0 (0.00%) |
| M46.36 | 2 (14.29%) | 1 (10.00%) | 1 (25.00%) |
| M46.54 | 1 (7.14%) | 1 (10.00%) | 0 (0.00%) |
| M47.12 | 9 (64.29%) | 6 (60.00%) | 3 (75.00%) |
| M47.86 | 1 (7.14%) | 1 (10.00%) | 0 (0.00%) |
| M60-M79 Soft tissue disorders | 2 (0.03%) | 2 (0.04%) | 0 (0.00%) |
| M60.09 | 1 (50.00%) | 1 (50.00%) | 0 (0.00%) |
| M72.65 | 1 (50.00%) | 1 (50.00%) | 0 (0.00%) |
| M80-M94 Osteopathies and chondropathies | 5 (0.08%) | 4 (0.08%) | 1 (0.08%) |
| M86.94 | 1 (20.00%) | 1 (25.00%) | 0 (0.00%) |
| M86.97 | 4 (80.00%) | 3 (75.00%) | 1 (100.00%) |
| N10-N16 Renal tubulo-interstitial diseases | 14 (0.22%) | 12 (0.23%) | 2 (0.15%) |
| N10 | 13 (92.86%) | 11 (91.67%) | 2 (100.00%) |
| N12 | 1 (7.14%) | 1 (8.33%) | 0 (0.00%) |
| N30-N39 Other diseases of urinary system | 4 (0.06%) | 3 (0.06%) | 1 (0.08%) |
| N39.0 | 4 (100.00%) | 3 (100.00%) | 1 (100.00%) |
| N70-N77 Inflammatory diseases of female pelvic organs | 1 (0.02%) | 1 (0.02%) | 0 (0.00%) |
| N71.1 | 1 (100.00%) | 1 (100.00%) | 0 (0.00%) |
| T80-T88 Complications of surgical and medical care, not elsewhere classified | 8 (0.12%) | 6 (0.12%) | 2 (0.15%) |
| T81.8 | 1 (12.50%) | 1 (16.67%) | 0 (0.00%) |
| T82.5 | 1 (12.50%) | 1 (16.67%) | 0 (0.00%) |
| T82.8 | 1 (12.50%) | 1 (16.67%) | 0 (0.00%) |
| T84.0 | 2 (25.00%) | 1 (16.67%) | 1 (50.00%) |
| T84.5 | 2 (25.00%) | 1 (16.67%) | 1 (50.00%) |
| T85.7 | 1 (12.50%) | 1 (16.67%) | 0 (0.00%) |

Abbreviations: ICD-10: International Classification of Diseases, 10th revision.

# **Supplementary Table S5.** Predictor coefficients and performance of the candidate models for serious infections in patients with rheumatoid arthritis prescribed with tocilizumab

| Model | 1 | 2 | 3 | 4 | 5 | 6 | 7 | 8 | 9 | 10 |
| --- | --- | --- | --- | --- | --- | --- | --- | --- | --- | --- |
| Number of patients in the model | 5199 | 5199 | 5199 | 5199 | 5199 | 5199 | 5199 | 5199 | 5199 | 5199 |
| Number of non-zero predictors | 17 | 16 | 16 | 17 | 21 | 7 | 6 | 9 | 6 | 9 |
| Coefficient |  |  |  |  |  |  |  |  |  |  |
| Intercept | -4.591 | -4.514 | -4.598 | -4.590 | -4.977 | -3.729 | -3.732 | -3.313 | -3.322 | -3.281 |
| Sex [Male] | 0.062 | 0.060 | 0.057 | 0.060 | 0.146 |  |  |  |  |  |
| Age, years | 0.013 | 0.014 | 0.013 | 0.013 | 0.015 | 0.007 | 0.007 |  |  |  |
| Age, categorised [40-49 years] |  |  |  |  |  |  |  |  |  |  |
| [50-59 years] |  |  |  |  |  |  |  |  |  |  |
| [60-69 years] |  |  |  |  |  |  |  |  |  |  |
| [≥70 years] |  |  |  |  |  |  |  |  |  |  |
| [≥65 years] |  |  |  |  |  |  |  | 0.080 | 0.080 |  |
| [≥75 years] |  |  |  |  |  |  |  |  |  | 0.024 |
| Duration of RA, categorised [≥10 years] |  |  |  |  | 0.047 |  |  |  |  |  |
| History of infections | 0.152 | 0.163 | 0.155 | 0.150 | 0.345 |  |  |  |  |  |
| History of serious infections within 1 year | 0.943 | 0.957 | 0.941 | 0.944 | 0.975 | 0.800 |  | 0.810 |  | 0.815 |
| History of serious infections, ever |  |  |  |  |  |  | 0.651 |  | 0.660 |  |
| Constipation | 0.311 | 0.319 | 0.313 | 0.310 | 0.343 | 0.188 | 0.176 | 0.202 | 0.190 | 0.207 |
| Liver diseases |  |  |  |  |  |  |  |  |  |  |
| Renal diseases | 0.031 | 0.028 | 0.029 | 0.029 | 0.053 |  |  |  |  |  |
| Respiratory diseases |  |  |  |  | -0.080 |  |  |  |  |  |
| Heart diseases | 0.330 | 0.334 | 0.329 | 0.328 | 0.340 | 0.293 | 0.271 | 0.310 | 0.288 | 0.316 |
| Vascular disease | 0.194 | 0.201 | 0.198 | 0.191 | 0.197 | 0.153 | 0.141 | 0.183 | 0.170 | 0.195 |
| Diverticulitis and related disorders | 0.781 | 0.767 | 0.772 | 0.779 | 0.876 | 0.278 | 0.195 | 0.309 | 0.222 | 0.311 |
| Amyloidosis | 0.053 | 0.051 | 0.050 | 0.053 | 0.216 |  |  |  |  |  |
| Gastrointestinal ulcers | 0.197 | 0.205 | 0.199 | 0.197 | 0.250 | 0.012 |  | 0.012 |  | 0.012 |
| Knee or hip prosthesis within 5 years | 0.130 |  |  | 0.129 | 0.282 |  |  |  |  |  |
| Knee or hip prosthesis, ever |  | 0.003 |  |  |  |  |  |  |  |  |
| Interstitial lung diseases within 5 years |  |  |  |  |  |  |  |  |  |  |
| Interstitial lung diseases, ever |  |  |  |  | -0.020 |  |  |  |  |  |
| Diabetes within 5 years |  |  |  | 0.079 |  |  |  |  |  |  |
| Diabetes ever | 0.061 | 0.069 | 0.062 |  | 0.095 |  |  |  |  |  |
| Myocardial infarction within 5 years |  |  |  |  | -0.217 |  |  |  |  |  |
| Myocardial infarction, ever |  |  |  |  |  |  |  |  |  |  |
| Chronic kidney disease within 5 years | 0.084 | 0.090 | 0.089 | 0.084 | 0.082 |  |  |  |  |  |
| Chronic kidney disease, ever |  |  |  |  |  |  |  |  |  |  |
| IBD within 5 years |  |  |  |  |  |  |  |  |  |  |
| IBD, ever |  |  |  |  |  |  |  |  |  |  |
| Malignancies^a^ within 10 years | 0.203 |  | 0.201 | 0.202 | 0.254 |  |  |  |  |  |
| Malignancies^a^, ever |  | 0.200 |  |  |  |  |  | 0.001 |  | 0.003 |
| Steroid use during the 1-week period |  |  |  |  |  |  |  |  |  |  |
| Steroid use during the 1-year period | 0.151 |  | 0.152 | 0.150 | 0.242 |  |  |  |  |  |
| Steroid use, categorised  [>0 to ≤5 mg/day] |  |  |  |  |  |  |  |  |  |  |
| Steroid use, categorised  [>5 mg/day] |  |  |  |  |  |  |  |  |  |  |
| MTX use during the 1-week period | -0.191 | -0.194 | -0.190 | -0.191 | -0.254 |  |  | -0.009 |  | -0.015 |
| Number of previous treatments with DMARDs, categorised  [>5] |  |  |  |  |  |  |  |  |  |  |
|  |  |  |  |  |  |  |  |  |  |  |
| Discrimination |  |  |  |  |  |  |  |  |  |  |
| Cross entropy, internal cross-validation (training set) | 0.1820 | 0.1821 | 0.1822 | 0.1823 | 0.1823 | 0.1852 | 0.1853 | 0.1857 | 0.1857 | 0.1858 |
| Cross entropy (test set) | 0.1488 | 0.1486 | 0.1489 | 0.1488 | 0.1483 | 0.1499 | 0.1504 | 0.1502 | 0.1507 | 0.1502 |
| AUC (test set) | 0.712 | 0.714 | 0.710 | 0.712 | 0.716 | 0.698 | 0.697 | 0.689 | 0.688 | 0.686 |
| Confidence interval AUC 95%^b^ | 0.649-0.774 | 0.654-0.775 | 0.648-0.772 | 0.650-0.774 | 0.655-0.778 | 0.628-0.767 | 0.628-0.765 | 0.617-0.762 | 0.616-0.760 | 0.610-0.761 |
|  |  |  |  |  |  |  |  |  |  |  |
| Accuracy at the 5% predicted risk in the test set |  |  |  |  |  |  |  |  |  |  |
| Sensitivity | 33/46 (72%) | 33/46 (72%) | 33/46 (72%) | 32/46 (70%) | 32/46 (70%) | 31/46 (67%) | 32/46 (70%) | 29/46 (63%) | 31/46 (67%) | 29/46 (63%) |
| Specificity | 802/1256 (64%) | 798/1256 (64%) | 799/1256 (64%) | 801/1256 (64%) | 813/1256 (65%) | 841/1256 (67%) | 836/1256 (67%) | 848/1256 (68%) | 855/1256 (68%) | 870/1256 (69%) |
| Positive predictive value | 33/487 (7%) | 33/491 (7%) | 33/490 (7%) | 32/487 (7%) | 32/475 (7%) | 31/446 (7%) | 32/452 (7%) | 29/437 (7%) | 31/432 (7%) | 29/415 (7%) |
| Negative predictive value | 802/815 (98%) | 798/811 (98%) | 799/812 (98%) | 801/815 (98%) | 813/827 (98%) | 841/856 (98%) | 836/850 (98%) | 848/865 (98%) | 855/870 (98%) | 870/887 (98%) |

Abbreviations: AUC: area under the receiver operating characteristic curve, DMARDs: disease-modifying antirheumatic drugs, IBD: inflammatory bowel diseases; MTX: methotrexate, RA: rheumatoid arthritis.

Note: The test set is a subset of the full analysis set (the latest 20% of eligible patients of the dataset) to evaluate the predictive performance of the models.

^a^ Only the patients without the active malignancy treatment at the index date.

^b^ 95% CI computed with 2000 bootstrap replicates.

# **Supplementary Table S6.** Equation for the developed model for serious infection risk in patients with rheumatoid arthritis (Model 1)

| Estimated risk of serious infections in rheumatoid arthritis patients, based on predictor values, $x$, can be obtained as: |
| --- |
| $\pi\left( x \right)=\frac{1}{1+e^{-g\left( x \right)}} ,$  where  $\pi\left( x \right)=Probability\left( infection \vert x \right)$  $g\left( x \right)= -4.591+0.062\cdot x_{1}+0.013\cdot x_{2}+0.152\cdot x_{3}+0.943\cdot x_{4}+0.311\cdot x_{5}+0.031\cdot x_{6}+0.330\cdot x_{7}+0.194\cdot x_{8}+0.781\cdot x_{9}+0.053\cdot x_{10}+0.197\cdot x_{11}+0.130\cdot x_{12}+0.061\cdot x_{13}+0.084\cdot x_{14}+0.203\cdot x_{15}+0.151\cdot x_{16}-0.191\cdot x_{17}$  $x_{1}$ – 1, if sex = Male; 0, otherwise  $x_{2}$ – Age, years  $x_{3}$ – 1, if history of infection (ever) = Yes; 0, otherwise  $x_{4}$ – 1, if history of previous serious infection (within 1 year) = Yes; 0, otherwise  $x_{5}$ – 1, if constipation (ever) = Yes; 0, otherwise  $x_{6}$ – 1, if renal disease (ever) = Yes; 0, otherwise  $x_{7}$ – 1, if heart disease (ever) = Yes; 0, otherwise  $x_{8}$ – 1, if vascular disease (ever) = Yes; 0, otherwise  $x_{9}$ – 1, if diverticulitis and related disorders (ever) = Yes; 0, otherwise  $x_{10}$ – 1, if amyloidosis (ever) = Yes; 0, otherwise  $x_{11}$ – 1, if gastrointestinal ulcers (ever) = Yes; 0, otherwise  $x_{12}$ – 1, if knee or hip prosthesis (within 5 years) = Yes; 0, otherwise  $x_{13}$ – 1, if diabetes, ever = Yes; 0, otherwise  $x_{14}$ – 1, if chronic kidney disease (within 5 years) = Yes; 0, otherwise  $x_{15}$ – 1, if malignancies (within 10 years) = Yes; 0, otherwise  $x_{16}$ – 1, if steroid use (within 1 year) = Yes; 0, otherwise  $x_{17}$ – 1, if methotrexate use (within 1 week) = Yes; 0, otherwise |

# **Supplementary Table S7.** Risk calculator of serious infections within a year in patients with rheumatoid arthritis treated with tocilizumab

See the Excel file under supplementary material for Supplementary Table S7.
